# Comparative Antinociceptive Evaluation of Hofmeisterin I and Analogues from *Hofmeisteria schaffneri* in Zebrafish and Mice

**DOI:** 10.1021/acsomega.6c01037

**Published:** 2026-04-11

**Authors:** Sebastián Martínez Flores, Gabriela García-Marín, María Guadalupe Martínez-Villarreal, Manuel López-Ortiz, Manuel Eduardo Rangel-Grimaldo, Simón Hernández Ortega, Myrna Déciga-Campos, Rachel Mata

**Affiliations:** † Departamento de Farmacia, Facultad de Química, 7180 Universidad Nacional Autónoma de México, Ciudad de México 04510, Mexico; ‡ Laboratorio de Síntesis Orgánica, UMIEZ Facultad de Estudios Superiores Zaragoza, 7180 Universidad Nacional Autónoma de México, Campus II, Ciudad de México 09230, Mexico; § Departamento de Productos Naturales y Laboratorio de Difracción de Rayos X, Instituto de Química, 7180 Universidad Nacional Autónoma de México, Ciudad de México 04510, México; ∥ Sección de Estudios de Posgrado e Investigación, Escuela Superior de Medicina, Instituto Politécnico Nacional, Ciudad de México 11340, Mexico

## Abstract

*Hofmeisteria schaffneri* (Asteraceae) is a medicinal plant used in Central Mexico for pain relief. Although thymol- and northymol-derived metabolites contribute to its antinociceptive activity, the low abundance of the latter has limited evaluation. An optimized synthetic route to hofmeisterin I (**1**) improved yields and reduced reaction times, enabling biological assessment. Hofmeisterin I (**1**) and derivatives were tested in zebrafish (*Danio rerio*) and murine formalin models. Halogenated derivatives were toxic and minimally active, whereas the acetylated analogue exhibited higher potency but limited safety. A new nitrogen-containing imide analogue, namely, 1-(2-(5-bromo-2-hydroxy-4-methylphenyl)-2-oxoethyl)­pyrrolidine-2,5-dione (**12**), exhibited improved efficacy and a safer profile. Mechanistic studies indicated the involvement of TRPV1, CB_2_, and PPARγ receptors, with modulation by the opioid and serotonergic systems. Collectively, these findings highlight compound **12** as a promising antinociceptive scaffold. X-ray analyses of **1** and **12** are also reported.

## Graphical abstract

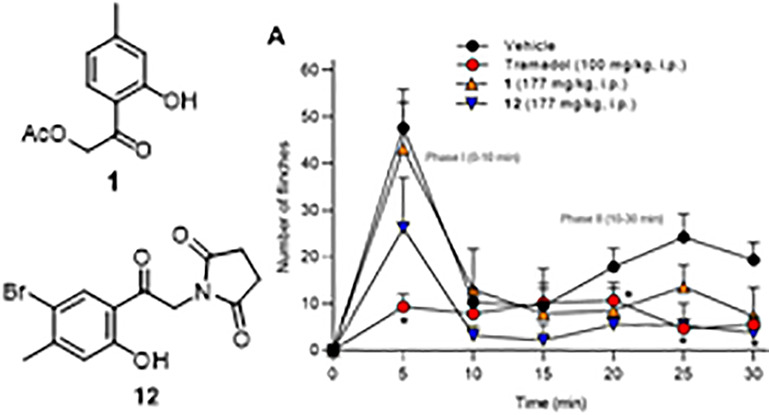

## 1 Introduction

*Hofmeisteria schaffneri* (A. Gray) R.M. King & H. Robinson (Asteraceae) is widely used in Central Mexico to treat stomach pain and other ailments. Phytochemical studies have identified thymol- and northymol-derived metabolites as the main contributors to its antinociceptive activity. Among them, hofmeisterin I (**1**), 8,9-epoxy-10-acetoxythymyl angelate (**2**), and hofmeisterin III (**3**) have been isolated (Figure 1), with compounds **2** and **3** accounting for most of the plant’s analgesic effect.^1,2^

**Figure 1:**
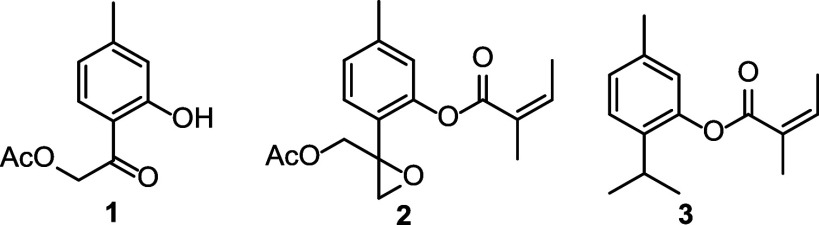
Selected compounds previously isolated from *Hofmeisteria schaffneri*.

Despite its potential pharmacological importance, hofmeisterin I (**1**) and other minor constituents have not been fully studied due to their low natural abundance. Conversely, a previous synthetic route to northymol **1** was reported, **2** but it proved inefficient for in vivo studies. From a pharmacological perspective, hofmeisterin I (**1**) may offer advantages over other active compounds from the plant because of its higher water solubility and the lack of PAINS^3^ associated with the angelic acid motif found in compounds **2** and **3**.

Therefore, the central hypothesis of this study is that optimizing the synthesis of hofmeisterin I (**1**) will not only generate enough material for biological testing but also enable exploration of structure–activity relationships to find derivatives with better antinociceptive effects. Accordingly, this work aimed to (i) develop an efficient synthetic route to hofmeisterin I (**1**), (ii) synthesize selected derivatives including halogenated, acetylated, and nitrogen-containing imide analogues, and (iii) evaluate their acute toxicity and antinociceptive activity using zebrafish (*Danio rerio* Hamilton-Buchanan) and murine formalin models.

## 2 Experimental Section

### 2.1 Chemicals and Reagents

All reagents and solvents were obtained from Merck (Merck, Darmstadt, Germany) and used without further purification. Analytical reagent (AR)- grade solvents for extraction, fractionation, and purification (acetone, methanol, hexane, ethyl acetate, chloroform, and dichloromethane) were purchased from J.T. Baker (Avantor, Radnor, PA, USA).

### 2.2 General Experimental Procedures

IR spectra were performed on a PerkinElmer Spectrum 400 FTIR/FIR spectrophotometer (PerkinElmer Inc., Waltham, MA, USA). 1D and 2D NMR spectra were recorded on a JEOL Eclipse (Peabody, MA, USA), a Bruker Avance III (Billerica, MA, USA), or a JEOL ECZ600R (Peabody, MA, USA) spectrometer at room temperature, operating at 300, 400, and 600 MHz for ^1^H and 75, 100, and 150 MHz for ^13^C, respectively. Residual CDCl_3_ (δ_H_ 7.26; δ_C_ 7.77) was used as the internal reference. High-resolution mass spectrometry (ESI-TOF) was conducted using an Agilent Technologies 6530 Accurate-Mass Q-TOF LC/MS equipment. X-ray analyses of compounds **1**, **9b**, and **12** were performed with a Bruker Smart Apex diffractometer (Billerica, MA, USA) with Mo radiation (λ = 0.71073 Å) at 173, 150, and 173 °K, respectively. Column chromatography (CC) was carried out on silica gel 60 (Merck, Darmstadt, Germany). Thin-layer chromatography (TLC) was performed on precoated Merck Silica Gel GF254 glass sheets (0.25 mm thickness) using multiple elution systems. Column chromatography was performed using silica gel 60 (Merck) and Sephadex LH-20 (Merck). Reaction progress was monitored by TLC; spots were visualized under UV light (254 nm) or with phosphomolybdic acid and vanillin. Melting points were recorded using the Fischer-Johns apparatus and were uncorrected.

### 2.3 Synthesis of Hofmeisterin I (**1**) and Analogs **9a**, **9b**, **10**, and **12**

#### 2.3.1 Synthesis of 1-(2-(Benzyloxy)-4-methylphenyl)­ethan-1-one (**5**)

Five grams (33.33 mmol) of 1-(2-hydroxy-4-methylphenyl)­ethan-1-one (**4**), prepared according to the procedure described by Bensari & Zaveri,^4^ were placed in a round-bottomed flask with 8.84 g (49.97 mmol, 1.5 equiv) of benzyl bromide, 9.11 g (66.63 mmol, 2 equiv) of K_2_CO_3_, and 25 mL of DMF.^5^ The mixture was heated at 80 °C for 3 h, then cooled to room temperature. Water was added, and the solid precipitate was collected and dried under vacuum. The raw product was recrystallized from ethanol to afford 6.2 g of white crystals (89%) of **5**, mp 55–56 °C. ^1^H NMR (400 MHz, CDCl_3_): δ 7.70 (d, *J* = 7.8 Hz, 1H), 7.49–7.31 (m, 5H), 6.87–6.79 (m, 2H), 5.15 (s, 2H), 2.57 (s, 3H), 2.38 (s, 3H). ^13^C NMR (101 MHz, CDCl_3_): δ 199.4, 158.4, 144.9, 136.4, 128.8, 128.3, 127.7, 126.1, 121.9, 113.6, 70.8, 32.3, 22.0. IR (FT-IR) ν_max_ (cm^–1^) 3305, 3066, 2948, 1659, 1603, 1241, 996. HRESIMS, *m*/*z* 241.12224 [M + H]^+^ (calcd for C_16_H_17_O_2_, 241.12285).

#### 2.3.2 Synthesis of 1-(2-(Benzyloxy)-5-bromo-4-methylphenyl)­ethan-1-one (**6a**)

In a round-bottomed flask, 1 eq. of **5** (20.83 mmol), 1.05 eq. of NBS, and AcOH (1 mL per gram of aromatic compound) were added. The reaction mixture was heated at 80 °C for 30 min, then cooled to room temperature. Water was added, and the precipitate was collected and dried under vacuum. The raw product was recrystallized from ethanol to afford 6.5 g of white crystals (98%) of **6a**, mp 72–73 °C. ^1^H NMR (400 MHz, CDCl_3_): δ 7.92 (s, 1H), 7.47–7.32 (m, 5H), 6.91 (s, 1H), 5.13 (s, 2H), 2.56 (s, 3H), 2.40 (s, 3H). ^13^C NMR (101 MHz, CDCl_3_): δ 197.8, 157.3, 144.0, 136.0, 134.1, 128.9, 128.5, 127.7, 116.2, 115.5, 71.2, 32.1, 23.7. IR (FT-IR) ν_max_ (cm^–1^) 3302, 3034, 2923, 1660, 1595, 1385, 1254, 1009, 688. HRESIMS, *m*/*z* 319.03237 [M + H]^+^ (calcd for C_16_H_16_O_2_Br, 319.03337).

#### 2.3.3 Synthesis of 1-(2-(Benzyloxy)-5-chloro-4-methylphenyl)­ethan-1-one (**6b**)

In a round-bottomed flask, 1 eq. of **5** (6.96 mmol), 1.05 eq. of NCS, and AcOH (1 mL per gram of aromatic compound) were added. The reaction mixture was heated at 80 °C for 30 min, then cooled to room temperature. Water was added, and the precipitated solid was collected and dried under vacuum. The raw product was recrystallized from ethanol to afford 1.6 g of white crystals of **6b** (84%), mp 76–78 °C. ^1^H NMR (300 MHz, CDCl_3_): δ 7.76 (s, 1H), 7.46–7.38 (m, 5H), 6.90 (s, 1H), 5.14 (s, 2H), 2.56 (s, 3H), 2.38 (s, 3H). ^13^C NMR (75 MHz, CDCl_3_): δ 197.9, 156.7, 142.1, 136.0, 130.8, 130.8, 128.9, 128.5, 127.7, 115.6, 71.2, 32.2, 20.8. IR (FT-IR) ν_max_ (cm^–1^) 3302, 3036, 2925, 1662, 1600, 1370, 1256, 1172, 978, 691. HRESIMS, *m*/*z* 275.08280 [M + H]^+^ (calcd for C_16_H_16_ClO_2_, 275.08388).

#### 2.3.4 Synthesis of 1-(2-(Benzyloxy)-5-bromo-4-methylphenyl)-2-bromoethan-1-one (**7a**)

In a round-bottomed flask, 17.46 mmol of methylketone **6a**, 1.05 eq. of NBS, and 10% mol of TsOH were added. The reaction mixture was triturated for 15 min, then heated at 80 °C for 15 min.^6^ Water was added, and the precipitated solid was collected and dried under vacuum. The raw product was recrystallized from ethanol to afford 5.71 g of white crystals of **7a** (82%), mp 76–78 °C. ^1^H NMR (400 MHz, CDCl_3_): δ 7.98 (s, 1H), 7.48–7.34 (m, 5H), 6.94 (s, 1H), 5.16 (s, 2H), 4.45 (s, 2H), 2.42 (s, 3H). ^13^C NMR (101 MHz, CDCl_3_): δ 190.9, 157.0, 145.3, 135.4, 135.0, 129.1, 128.9, 128.0, 124.4, 116.6, 115.3, 71.6, 37.0, 23.8. IR (FT-IR) ν_max_ (cm^–1^) 3308, 3037, 2907, 1660, 1593, 1383, 1229, 1024, 850, 728. HRESIMS, *m*/*z* 396.94435 [M + H]^+^ (calcd for C_16_H_15_O_2_Br_2_, 396.94388).

#### 2.3.5 Synthesis of 1-(2-(Benzyloxy)-5-chloro-4-methylphenyl)-2-bromoethan-1-one (**7b**)

In a round-bottomed flask, 5.1 mmol of methylketone **6b**, 1.05 eq. of NBS, and 10% mol of TsOH were added. The reaction mixture was triturated for 15 min and then heated at 80 °C for 15 min.^6^ Water was added, and the precipitated solid was collected and dried under vacuum. The raw product was recrystallized from ethanol to afford 1.6 g of white crystals of **7b** (89%), mp 80–82 °C. ^1^H NMR (300 MHz, CDCl_3_): δ 7.82 (s, 1H), 7.46–7.41 (m, 5H), 6.93 (s, 1H), 5.17 (s, 2H), 4.46 (s, 2H), 2.40 (s, 3H). ^13^C NMR (75 MHz, CDCl_3_): δ 191.1, 156.4, 143.5, 135.5, 131.8, 129.1, 128.9, 128.0, 124.1, 115.4, 71.6, 37.1, 21.0. IR (FT-IR) ν_max_ (cm^–1^) 3339, 3035, 2956, 1677, 1597, 1373, 1261, 1174, 996, 693. HRESIMS, *m*/*z* 352.99493 [M + H]^+^ (calcd for C_16_H_15_O_4_BrCl, 352.99439).

#### 2.3.6 Synthesis of 2-(2-(Benzyloxy)-5-bromo-4-methylphenyl)-2-oxoethyl acetate (**8a**)

In a round-bottomed flask 13.57 mmol of 2-bromoketone **7a**, 5 eq. of NaOAc and DMF (5 mL per gram of 2-bromoketone) were placed. The reaction mixture was heated at 80 °C for 1 h, then cooled to room temperature. Water was added, and the precipitate was collected and dried under vacuum. The raw product was recrystallized from ethanol to afford 4 g of slightly yellow crystals of **8a** (78%), mp 86–88 °C. ^1^H NMR (400 MHz, CDCl_3_): δ 8.08 (s, 1H), 7.47–7.33 (m, 5H), 6.93 (s, 1H), 5.15 (s, 2H), 5.12 (s, 2H), 2.41 (s, 3H), 2.16 (s, 3H). ^13^C NMR (101 MHz, CDCl_3_): δ 191.5, 170.6, 157.8, 145.4, 135.4, 134.5, 129.1, 128.8, 128.0, 124.1, 116.7, 115.3, 71.4, 70.0, 23.8, 20.7. IR (FT-IR) ν_max_ (cm^–1^) 3458, 3043, 2952, 1735, 1682, 1595, 1375, 1375, 1233, 1000, 728. HRESIMS, *m*/*z* 377.03837 [M + H]^+^ (calcd for C_18_H_18_O_4_Br, 377.03885).

#### 2.3.7 Synthesis of 2-(2-(Benzyloxy)-5-chloro-4-methylphenyl)-2-oxoethyl acetate (**8b**)

In a round-bottomed flask, 3.96 mmol of 2-bromoketone **7b**, 5 eq. of NaOAc and DMF (5 mL per gram of 2-bromoketone) were placed. The reaction mixture was heated at 80 °C for 1 h, then cooled to room temperature. Water was added, and the precipitate was collected and dried under vacuum. The raw product was recrystallized from ethanol to afford 0.85 g of slightly yellow crystals of **8b** (63%), mp 90–92 °C. ^1^H NMR (400 MHz, CDCl_3_): δ 7.91 (s, 1H), 7.49–7.36 (m, 5H), 6.93 (s, 1H), 5.15 (s, 2H), 5.12 (s, 2H), 2.39 (s, 3H), 2.17 (s, 3H). ^13^C NMR (101 MHz, CDCl_3_): δ 191.5, 170.6, 157.1, 143.6, 135.4, 131.2, 129.0, 128.8, 128.0, 127.3, 123.8, 115.3, 71.5, 70.1, 21.0, 20.7. IR (FT-IR) ν_max_ (cm^–1^) 3340, 3035, 1677, 1597, 1373, 1261, 1174, 996, 693. HRESIMS, *m*/*z* 333.08823 [M + H]^+^ (calcd for C_18_H_18_O_4_Cl, 333.08936).

#### 2.3.8 Synthesis of 1-(2-(2-(Benzyloxy)-5-bromo-4-methylphenyl)-2-oxoethyl)­pyrrolidine-2,5-dione (**11**)

In a round-bottom flask, 796 mg (2 mmol) of **7a**, 297.27 mg (3 mmol) of *N*-succinimide, 552 mg (4 mmol) of K_2_CO_3_, and 20 mL of MeCN were mixed. The reaction mixture was heated at 80 °C for 2 h. The solvent was filtered through a Celite pad and evaporated under reduced pressure. The residue was recrystallized from ethanol to afford 550 mg of **11** as a slightly orange solid (66%), mp 88–90 °C. ^1^H NMR (400 MHz, CDCl_3_): δ 8.06 (s, 1H), 7.47–7.34 (m, 5H), 6.95 (s, 1H), 5.19 (s, 2H), 4.79 (s, 2H), 2.79 (s, 4H), 2.42 (s, 3H). ^13^C NMR (101 MHz, CDCl_3_): δ 189.4, 176.9, 158.0, 145.8, 135.4, 134.9, 129.1, 128.8, 128.0, 124.0, 116.7, 115.4, 71.5, 49.2, 28.4, 23.9. IR (FT-IR) ν_max_ (cm^–1^) 2943, 1704, 1683, 1592, 1375, 1259, 1177, 1001, 671. HRESIMS, *m*/*z* 416.04939 [M + H]^+^ (calcd for C_20_H_19_BrO_4_, 416.04975).

#### 2.3.9 Synthesis of 2-(5-Bromo-2-hydroxy-4-methylphenyl)-2-oxoethyl acetate (**9a**)

In a hydrogenation flask, 6.89 mmol of benzyl ether **8a**, 10% (w/w) of PtO_2_, and AcOEt (10 mL per gram of benzyl ether) were placed under a stream of anhydrous N_2_. The flask was then pressurized with H_2_ (60 bar) and shaken until the reaction was complete (2–3 h). After the reaction was completed, the catalyst was removed by filtration over Celite, and the solvent was evaporated under reduced pressure. The residue was purified by CC [silica gel, heptane-EtOAc (9:1)] to afford 1.9 g of **9a** as a white crystalline solid (98%), mp 86–87 °C. ^1^H NMR (600 MHz, CDCl_3_): δ 11.49 (s, 1H), 7.74 (s, 1H), 6.91 (s, 1H), 5.29 (s, 2H), 2.39 (s, 3H), 2.24 (s, 3H). ^13^C NMR (75 MHz, CDCl_3_): δ 196.2, 170.4, 161.4, 148.3, 131.5, 120.8, 116.7, 113.8, 64.8, 23.9, 20.6. IR (FT-IR) ν_max_ 3480, 3069, 2947, 1748, 1655, 1621, 1422, 1228, 1085, 944, 742. HRESIMS, *m*/*z* 286.99104 [M + H]^+^ (calcd for C_11_H_12_O_4_Br, 286.99190).

#### 2.3.10 Synthesis of 2-(5-Chloro-2-hydroxy-4-methylphenyl)-2-oxoethyl acetate (**9b**)

In a hydrogenation flask, 2.5 mmol of benzyl ether 8b, 10% (w/w) of PtO_2_, and AcOEt (10 mL per gram of benzyl ether) were placed under a stream of anhydrous N_2_. The flask was then pressurized with H_2_ (60 bar) and shaken until the reaction was complete (2–3 h). After the reaction was complete, the catalyst was removed by filtration over Celite, and the solvent was evaporated under reduced pressure. The residue was purified by recrystallization from ethanol to afford 0.520 g of **9b** as a white crystalline solid. (86%), mp 88–89 °C. ^1^H NMR (600 MHz, CDCl_3_): δ 11.48 (s, 1H), 7.56 (s, 1H), 6.90 (s, 1H), 5.29 (s, 2H), 2.38 (s, 3H), 2.24 (s, 3H). ^13^C NMR (150 MHz, CDCl_3_): 196.3, 170.4, 160.9, 146.6, 128.1, 124.8, 120.9, 116.1, 64.8, 21.1, 20.6. IR (FT-IR) ν_max_ (cm^–1^) 3476, 3136, 3081, 2926, 1743, 1671, 1424, 1235, 1174, 1029, 761_._ HRESIMS, *m*/*z* 243.04192 [M + H]^+^ (calcd for C_11_H_12_O_4_Cl, 243.04241).

#### 2.3.11 Synthesis of 1-(2-(5-Bromo-2-hydroxy-4-methylphenyl)-2-oxoethyl)­pyrrolidine-2,5-dione (**12**)

In a hydrogenation flask, 0.84 mmol of benzyl ether **11**, 10% (w/w) of PtO_2_, and AcOEt (10 mL per gram of benzyl ether) were placed under a stream of anhydrous N_2_. The flask was then pressurized with H_2_ (60 bar) and shaken until the reaction was complete (2–3 h). When the reaction was complete, the catalyst was removed by filtration over Celite, and the solvent was evaporated under reduced pressure. The residue was purified by recrystallization from ethyl acetate to afford 220 mg of **12** (80%), mp 87–88 °C. ^1^H NMR (400 MHz, CDCl_3_): δ 11.29 (s, 1H), 7.86 (s, 1H), 6.90 (s, 1H), 4.91 (s, 2H), 2.87 (s, 4H), 2.40 (s, 3H). ^13^C NMR (101 MHz, CDCl_3_): δ 194.3, 176.6, 161.4, 148.6, 132.0, 120.7, 117.1, 114.1, 44.0, 28.5, 23.9. IR (FT-IR) ν_max_ (cm^–1^) 3059, 2939, 1709, 1651, 1422, 1171, 883, 779, 659. HRESIMS, *m*/*z* 326.00319 [M + H]^+^ (calcd for C_13_H_13_BrNO_4_, 326.00280).

#### 2.3.12 Synthesis of Hofmeisterin (**1**)

In a hydrogenation flask, 1.8 g of **8a**, 3.6 g of Pd/C (10%), and 90 mL of acetic acid were added under a stream of anhydrous N_2_. The flask was then pressurized with H_2_ (60 bar) and shaken at 60 °C for 18 h. The catalyst was removed by filtration over Celite, and the solvent was evaporated under reduced pressure. The residue was purified by CC [silica gel, heptane-EtOAc (9:1)] to yield 800 mg of **1** as white crystals (61%), mp 86–87 °C. ^1^H NMR (600 MHz, CDCl_3_): δ 11.66 (s, 1H), 7.49 (d, *J* = 8.2 Hz, 1H), 6.81 (s, 1H), 6.72 (dd, *J* = 8.3, 1.6 Hz, 1H), 5.32 (s, 2H), 2.35 (s, 3H), 2.24 (s, 3H). ^13^C NMR (150 MHz, CDCl_3_): δ 196.8, 170.5, 162.6, 149.0, 120.7, 118.9, 115.1, 64.9, 22.2, 20.6. IR (FT-IR) ν_max_ (cm^–1^) 3094, 2948, 1744, 1651, 1428, 1382, 1275, 1203, 1074, 977, 751. HRESIMS, *m*/*z* 209.08146 [M + H]^+^ (calcd for C_11_H_13_O_4_, 209.08138).

#### 2.3.13 Synthesis of 2-(2-Acetoxy-4-methylphenyl)-2-oxoethyl acetate (**10**)

In a round-bottom flask, 42 mg (0.2 mmol) of **1** and 35.6 mg (0.352 mmol) of EtN_3_ were dissolved in DCM (0.2 M). At 0 °C, 21.62 mg (0.275 mmol) of acyl chloride was added. The reaction mixture was stirred for 30 min. The solvent was removed under reduced pressure. The residue was purified by column CC [silica gel, heptane-EtOAc (9:1)] to yield 50 mg of **11** as a white solid (99%), mp 46–48 °C. ^1^H NMR (600 MHz, CDCl_3_): δ 7.71 (d, *J* = 7.9 Hz, 1H), 7.13 (dd, *J* = 8.1, 1.6 Hz, 1H), 6.97 (s, 1H), 5.13 (s, 2H), 2.40 (s, 3H), 2.35 (s, 3H), 2.18 (s, 3H). ^13^C NMR (150 MHz, CDCl_3_): δ 191.7, 170.5, 169.3, 149.5, 145.7, 129.8, 127.2, 125.2, 124.5, 67.6, 21.6, 21.3, 20.6. IR (FT-IR) ν_max_ (cm^–1^) 3013, 2927, 1763, 1740, 1692, 1610, 1410, 1374, 1192, 1119, 895. HRESIMS, 251.09119 *m*/*z* [M + H]^+^ (C_11_H_15_O_4_, 251.09195).

### 2.4 X-ray Crystal Structure Analysis of 1, 9b, and 12

#### 2.4.1 Hoffmeisterin (**1**)

In the case of **1**, crystals suitable for X-ray analysis were obtained by recrystallization from ethanol. A colorless crystal, approximately 0.451 × 0.186 × 0.124 mm^3^, was mounted on a glass fiber. The crystallographic information has been deposited in the Cambridge Crystallographic Data Centre; deposition number: 2524512.

Crystal data for **1**: C_11_H_11_ClO_4_, MW 208.21, triclinic, space group P-1 with unit cell parameters: *a* = 7.7264(3) Å, *b* = 7.8747(3) Å, *c* = 9.92927(6) Å, α = 104.5840(10)°, β = 108.3379(10)°, γ = 99.1070°, *Z* = 2, *T*= 173(2) K, Volume: 504.25(3) Å^3^, F(000): 220, Density (calc.): 1.371 Mg/m^3^. Intensity data were collected in the range of 2.433 to 27.533° using a ω scan. 22,005 reflections collected, 2282 independent reflections [*R*(int)= 0.0331] were considered, observed, and used in the calculations. The final R1 indices were 0.0518 [*I* > 2σ­(*I*)]. The final w*R*_2_ value was 0.1410 [*I* > 2σ­(*I*)], with a data/restraints/parameters ratio of 2282/1/141. The final R1 values were 0.0639 (all data). The final w*R*_2_ values were 0.1565 (all data).

#### 2.4.2 2-(5-Chloro-2-hydroxy-4-methylphenyl)-2-oxoethyl acetate (**9b**)

In the case of **9b**, crystals suitable for X-ray analysis were obtained by recrystallization from ethanol. A colorless crystal, approximately 0.397 × 0.235 × 0.206 mm^3^, was mounted on a glass fiber. The crystallographic information has been deposited in the Cambridge Crystallographic Data Centre; deposition number 2524512.

Crystal data for **9b**: C_11_H_11_ClO_4_, MW 242.65, monoclinic, space group P21/n with unit cell parameters: *a* = 5.6964(4) Å, *b* = 24.8887(19) Å, *c* = 7.9832(6) Å, α = 90°, β = 101.401(2)°, γ = 90°, *Z* = 4, T = 150(2) K, Volume: 1109.49(14) Å^3^, F(000): 504, Density (calc.): 1.453 Mg/m^3^. Intensity data were collected in the range of 2.728 to 26.371° using a ω scan. 28,226 reflections collected, 2162 independent reflections [*R*(int)= 0.0947] were considered, observed, and used in the calculations. The final R1 indices were 0.0759 [*I* > 2σ­(*I*)]. The final w*R*_2_ value was 0.1115 [*I* > 2σ­(*I*)], with a data/restraints/parameters ratio of 2162/0/150. The final R1 values were 0.0777 (all data). The final w*R*_2_ values were 0.1123 (all data).

#### 2.4.3 1-(2-(5-Bromo-2-hydroxy-4-methylphenyl)-2-oxoethyl)­pyrrolidine-2,5-dione (**12**)

In the case of **12**, crystals suitable for X-ray analysis were obtained by recrystallization from ethyl acetate. A colorless crystal, approximately 0.371 × 0.343 × 0.292 mm^3^, was mounted on a glass fiber. The crystallographic information has been deposited in the Cambridge Crystallographic Data Centre; deposition number 2524513.

Crystal data for **12**: C_13_H_12_BrNO_4_, MW 326.15, monoclinic, space group P21/*n* with unit cell parameters: *a* = 9.9638(2) Å, *b* = 5.99970(10) Å, *c* = 22.0660(5) Å, α = 90°, β = 101.5070 (2)°, γ = 90°, *Z* = 4, *T* = 173(2) K, Volume: 1292.59 (14) Å^3^, F(000): 656, Density (calc.): 1.676 Mg/m^3^. Intensity data were collected in the range of 2.454 to 27.492° using a ω scan. 32121 reflections collected, 2902 independent reflections [*R*(int)= 0.0251] were considered, observed, and used in the calculations. The final R1 indices were 0.0200 [*I* > 2σ­(*I*)]. The final w*R*_2_ value was 0.0534 [*I* > 2σ­(*I*)], with a data/restraints/parameters ratio of 2902/1/176. The final R1 values were 0.0209 (all data). The final w*R*_2_ values were 0.0539 (all data).

### 2.5 Biological Testing

#### 2.5.1 Animals

To screen for antinociceptive activity, we used adult male and female zebrafish (500–900 mg; 2.0 ± 0.5 cm) obtained from a local commercial distributor in Mérida, Yucatán. They were housed in aerated tanks (90 × 40 × 37 cm) equipped with activated carbon filters and air pumps in a natural environment at 33–35 °C for at least 6 weeks before the experiments. The aquarium water temperature was 27.5 ± 0.5 °C, pH was 7 ± 0.5, and the light/dark cycle was 12 h. The dissolved oxygen concentration was 6 ± 0.1 mg/L, the total ammonia concentration was <0.01 mg/L, the total hardness was 6 mg/L, and the alkalinity was 22 mg/L CaCO_3_, according to established standards for zebrafish husbandry. Zebrafish were fed ad libitum with essential flakes for tropical fish (Bioma SA. de CV. Mexico).^7,8^ Six-week-old male CD-1 mice (25–40 g) were obtained from Círculo ADN, S.A. de C.V. and housed at 25 °C under a 12 h light/dark cycle with ad libitum access to water and Lab Diet 5001 pellets. Mice were randomly assigned to experimental groups (*n* = 6) and euthanized in a CO_2_ chamber after experimentation.

All procedures complied with the Mexican Official Norm for Animal Care and Handling (NOM-062-ZOO-1999), ARRIVE guidelines, the UK Animals (Scientific Procedures) Act 1986, EU Directive 2010/63/EU, and the U.S. National Research Council’s Guide for the Care and Use of Laboratory Animals. The study protocol was approved by the institutional committee (Approval No. SIP-2023–1277; CICUAL-FQUNAM). All efforts were made to minimize distress and reduce the number of animals used.

#### 2.5.2 Acute Oral Toxicity in Zebrafish

The acute toxicity study was conducted in adult zebrafish in accordance with OECD Test Guideline 203.^9^ The synthesized compounds were dissolved in 0.1% DMSO and tested at concentrations of 10, 30, 56.2, and 100 mg/L to determine the median lethal concentration (LC_50_). Each concentration group consisted of eight fish. Mortality was monitored at 24, 48, and 96 h. LC_50_ values were calculated based on mortality observed across the tested concentration range.

#### 2.5.3 Antinociceptive Effect in *Danio rerio*

The antinociceptive assay was conducted according to established protocols.^10,11^ Fish were exposed to logarithmic concentrations of the extract or synthesized compounds for 30 min before receiving the alogenic agent (10 μL of 5% acetic acid or 1.5% formalin), injected intramuscularly between the dorsal and caudal fins. After injection, animals were placed individually in a glass dish (20 × 15 cm) divided into eight quadrants. Locomotor activity, quantified as the number of line crossings, was recorded over a 40 min period.^12^ Following the toxicity assessment, the synthesized compounds (**1**, **9a**, **9b**, **10**, and **12**) were evaluated preliminarily at 3, 10, or 30 mg/L on 5% acetic acid, depending on toxicity. To confirm the antinociceptive activity, compounds **1** (10 or 30 mg/L) and **12** (30 or 100 mg/L) were evaluated using 5% acetic acid or 1.5% formalin. All treatments were prepared in 0.1% DMSO and added directly to the aquarium water. Tramadol (30 mg/L) was used as a positive control.

#### 2.5.4 Antinociceptive Effect on Mice

The nociceptive effect of formalin was assessed by flinching/shaking of the injected paw. In the first set of experiments, **1** and **12** were evaluated at 100, 177, and 300 mg/kg. The compounds were administered intraperitoneally to the different groups 30 min before subcutaneous injection of 1.5% formalin (20 μL) into the dorsal right hind paw. The number of flinches in the right injected paw was counted every 5 min for 30 min)^13^ Based on the zebrafish screening, two compounds (**1** and **12**) with potential antinociceptive activity were selected for evaluation in the mouse formalin test. Nociception was induced by subcutaneous injection of 1.5% formalin (20 μL) into the right hind paw, producing localized inflammation and pain. The nociceptive response was quantified as paw flinching or shaking and recorded at 5 min intervals over 30 min.13. Compounds **1** and **12** were administered intraperitoneally at 100, 177, and 300 mg/kg, 30 min prior to formalin injection. To explore potential mechanisms underlying the antinociceptive effect of **12** , mice were pretreated with the following antagonists: naloxone (1 mg/kg, opioid receptor antagonist), ketanserin (1 mg/kg, 5-HT_2_A/5-HT_4_ antagonist), capsazepine (3 mg/kg, TRPV1 antagonist), and AM630 (1 mg/kg, CB_2_ antagonist). Antagonists were administered 15 min before 10 (177 mg/kg, *i.p.*). Thirty minutes after administration, 1.5% formalin was injected, and flinches were quantified as described above.

### 2.6 Molecular Docking

Minimum-energy structures were built with Spartan’10 software (Wave function, Inc., Irvine, CA, USA). The minimized structures for docking simulations were prepared using the Autodock Tools package v1.5.6 (ADT, http://mgltools.scripps.edu/). For metabolites, the addition of Gasteiger charges and number of torsions was set, and nonpolar hydrogens were merged. The crystallographic structures of 5-HT_2B_,^14^ 5-HT_2C_,^15^ and μ-opioid^16^ receptors were obtained from the Protein Data Bank (pdb codes 5TVN, 6BQH, and 5C1M, respectively). 5-HT_2B_ receptor was cocrystallized with LSD; 5-HT_2C_ receptor with ketanserin; and μ-opiod receptor with BU72. The 5-HT_2A_ ^17^ receptor obtained by homology was retrieved from the Supporting Information of the paper by McRobb and collaborators.^16^ For the receptors, polar hydrogens and Kollman charges were added, and solvation parameters were assigned by default. Molecular docking was performed using AutoDock Vina 1.1.2.45. First, a blind docking was performed to identify the common interaction site between the metabolites and the receptors. The search space for this preliminary docking was defined as a 60 × 60 × 60 Å box centered on the macromolecule. Next, a refined docking was performed with a smaller search space (20 × 20 × 20 Å), using the lower-state pose from the blind docking as the grid center. The conformational states from the docking simulations were analyzed using AutoDock Tools and Maestro, which also identified hydrogen-bonding and van der Waals interactions between the receptors and ligands. The predicted docked complexes (protein–ligand) corresponded to the conformations with the lowest binding energy. The docking protocol was validated by reproducing the binding modes of each cocrystallized compound with each receptor. RMSD values less than 2 Å indicate a correct bound-structure prediction. Preparation of the figures was accomplished with the PyMOL visualization tool (PyMOL Molecular Graphics System v1.7.4, Schrödinger, New York, NY, USA)^18,19^ and Maestro 11.8.012 (Schrödinger).^20,21^

## 3 Results and Discussion

### 3.1 Synthesis of Hofmeisterin (**1**) and Analogs

An improved synthetic route to hofmeisterin I (**1**) was developed (Scheme 1) was developed. The initial benzylation step was shortened from 1 week to 3 h, and two halogenation steps (iii and iv in Scheme 1) were added, thereby boosting the overall yield and reducing reaction time. In intermediates **7a** and **7b**, the bromide was replaced with an acetoxy group using sodium acetate in DMF at 80 °C, producing **8a** and **8b**, respectively. Attempts to perform simultaneous dehalogenation and hydrogenolysis with various catalysts failed. PtO_2_ at room temperature selectively produced hydrogenolysis products **9a** and **9b** (Scheme 1). Follow-up catalytic dehalogenation of **9a** over Pd/C (10%) in acetic acid at 60 °C for 18 h yielded hofmeisterin I (**1**) in 61% (Scheme 2).

**Scheme 1:**
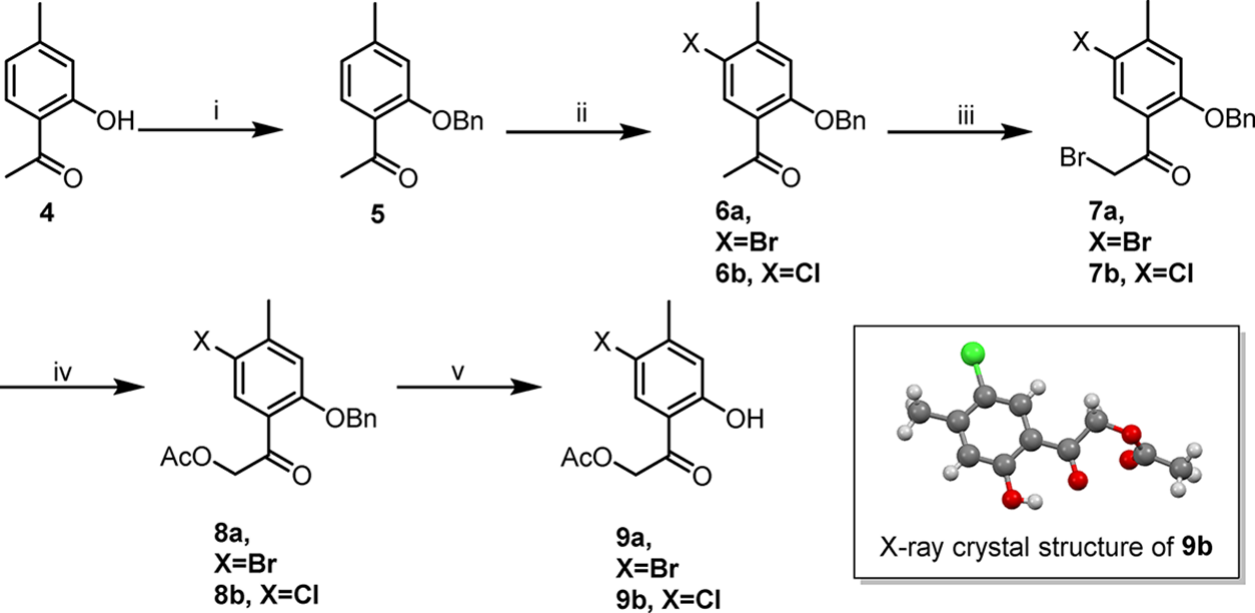
Synthesis Pathway of Hofmeisterin and Analogues^a^,^b^,^c^,^d^,^e^

a (i) BnBr, K_2_CO_3_, DMF, 80 °C, 3 h, 89%.
b (ii) NBS or NCS, AcOH, 80 °C, 30 min, 84–98%.
c (iii) NBS, TsOH, 80 °C, 15 min, 82–89%.
d (iv) AcONa, DMF, 80 °C, 1 h, 63–78%.
e (v) H_2_ (60 lb/inch^2^), PtO_2_, AcOEt, 2 h, 86–98%.

**Scheme 2:**
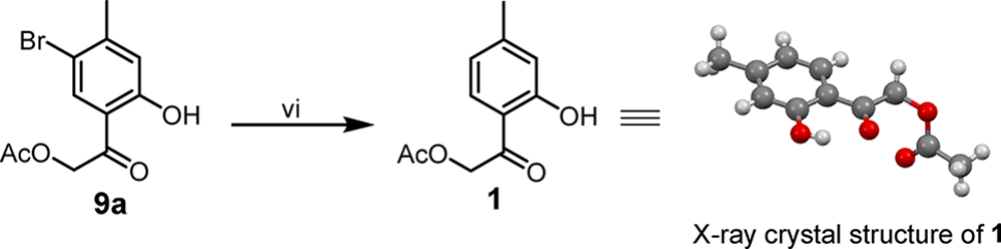
Dehalogenation of **9a** ^a^

a (vi) H_2_ (60 lb/inch^2^), Pd/c (10%), AcOH, 60 °C, 18 h, 61%.

The acetylated derivative **10** was prepared based on previous findings that esterified thymol derivatives exhibit enhanced antinociceptive activity compared with thymol (Scheme 3). Additionally, a nitrogen-containing imide analogue (**12**) was synthesized to potentially improve pharmacokinetic and bioavailability properties (Scheme 4).^22^ The halogenated derivatives were included to evaluate the effects of increased lipophilicity and electronic modulation on activity and toxicity, and because they were available in good amounts as reaction intermediates.

**Scheme 3:**
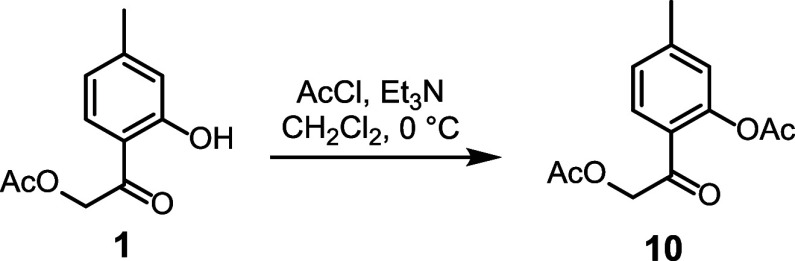
Synthesis of Acetyl Derivative **10**

**Scheme 4:**
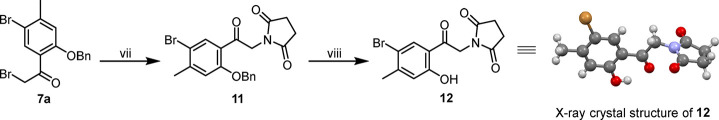
Synthesis Pathway of a Nitrogen-Containing Derivative^a^,^b^

a (vii) *N*-succinimide, K_2_CO_3_, MeCN, 80 °C, 2 h, 66%.
b (viii) H_2_ (60 lb/inch^2^), PtO_2_, AcOEt, 2 h, 80%.

### 3.2 Acute Toxicity in Zebrafish

Acute toxicity was assessed in zebrafish using OECD Test Guideline 203. Zebrafish are a cost-effective vertebrate model with ∼70% genomic homology to humans and comparable organ-system development. Compounds **1** and **12** were safe at concentrations up to 100 mg/L. The halogenated derivatives **9a** and **9b** exhibited higher toxicity (LC_50_:14.14–21.21 mg/L), and the acetylated derivative **10** showed intermediate toxicity (LC_50_:39.7 mg/L). These results highlight the importance of balancing efficacy and safety when selecting derivatives.

### 3.3 Antinociceptive Effect in Zebrafish

The antinociceptive activity was initially evaluated by measuring the reduction of locomotor activity in response to nociceptive stimuli (acetic acid) (Figure 2). Tramadol (30 mg/mL) was used as a positive control. Halogenated derivatives **9a** and **9b** showed minimal activity and caused toxicity at higher concentrations in the acetic acid model, which ruled out further investigation. The acetylated derivative **10** was more potent than **1** in this model, but, as before, safety concerns limited its use. These preliminary results led us to test the active compounds **1** and **12** in the same model, using formalin as the algogenic agent (Figure 3). In this case, both compounds showed significant antinociceptive effects while remaining safe, supporting their further evaluation in mice.^23^ Compound **12** exhibited a concentration-dependent antinociceptive effect.

**Figure 2:**
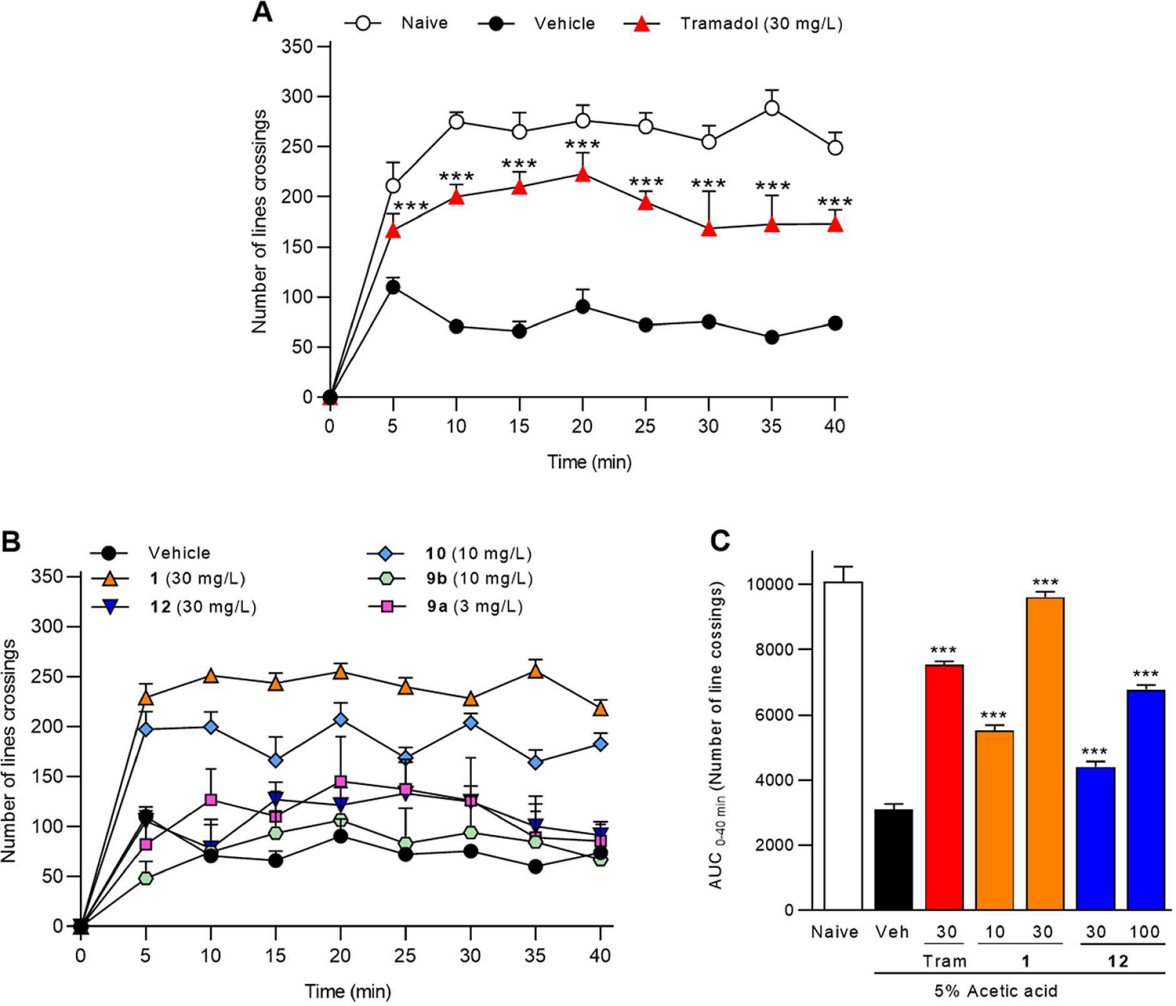
Experimental nociception test in *Danio rerio* induced by acetic acid. Nociceptive behavior was measured as the number of line crossings over a 40 min period, divided into 5 min intervals. Basal activity was evaluated in naive fish (normal behavior), and nociception was induced by intraperitoneal injection of 5% acetic acid (10 μL). Vehicle and tramadol (30 mg/L, positive control) are shown in panel A. The synthesized compounds (**1** at 30 mg/L; 10 at 10 mg/L; **9b** at 10 mg/L; **12** at 30 mg/L; **9a** at 3 mg/L) are presented in panel B. All treatments were administered 30 min before acetic acid injection. AUC values of **1** (10 and 30 mg/L) and **12** (30 and 100 mg/L) are shown in panel C. Data represent mean ± SEM (*n* = 8 per group). *p < 0.01 indicates significant differences versus vehicle (one-way ANOVA followed by Tukey’s test).

**Figure 3:**
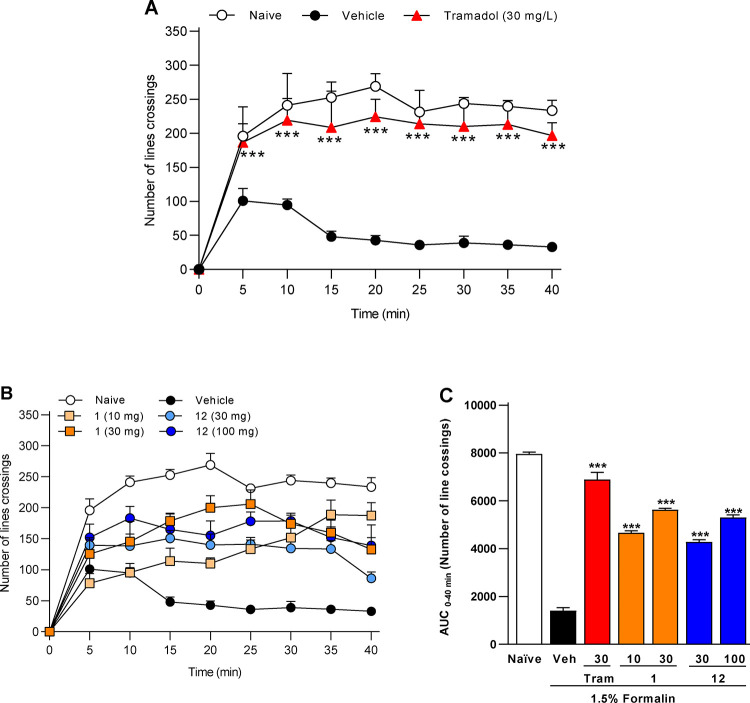
Experimental nociception test in *Danio rerio* induced by formalin. Nociceptive behavior was measured as the number of line crossings over a 40 min period, divided into 5 min intervals. Basal activity was assessed in naive fish exhibiting normal behavior, and nociception was triggered by intraperitoneal injection of 1.5% formalin (10 μL). Vehicle and tramadol (30 mg/L, positive control) are shown in panel A. The synthesized compounds (**1** at 10 or 30 mg/L; **12** over time in panel B, with AUC values shown in panel C. All treatments were administered 30 min before formalin injection. Data are presented as mean ± SEM (*n* = 8 per group). *p < 0.01 indicates significant differences compared to vehicle (one-way ANOVA followed by Tukey’s test).

### 3.4 Antinociceptive Effect on Mice

Zebrafish is a good model for preliminary screening of antinociceptive action; however, these results must be confirmed in murine models. Therefore, compounds **1** and **12** were evaluated using the 1.5% formalin test in mice, which allows differentiation between neurogenic (phase I, 0–10 min) and inflammatory (phase II, 10–30 min) nociceptive responses (Figure 4). The early phase is primarily attributed to activation of C fibers following peripheral stimulation, whereas the late phase is associated with peripheral inflammatory responses and functional changes in the dorsal horn of the spinal cord. These central sensitization processes are thought to be initiated by C-fiber discharge during the early phase.^24^ In this model, flinching and licking behaviors are well-established indicators of nociception. Tramadol (100 mg/kg, *i.p*.) was used as a positive reference drug. Figure 4A shows the nociceptive response to intraplantar formalin injections in vehicle-treated mice. A marked increase in flinching behavior was observed during both phase I and phase II.

**Figure 4:**
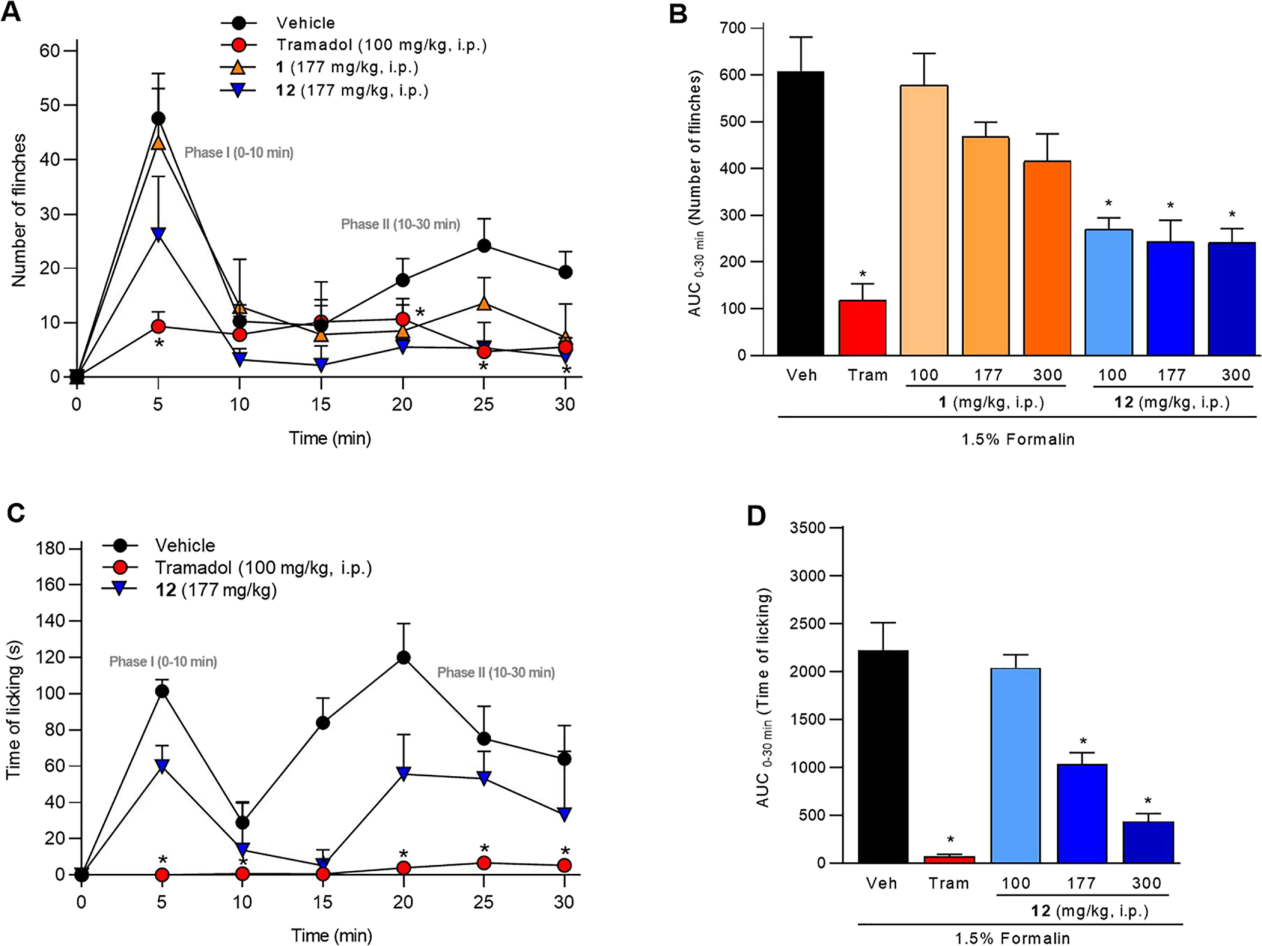
Experimental nociception test in mice induced by formalin. Nociceptive behavior was quantified as the number of flinches and time of licking over a 30 min period, divided into 5 min intervals. Nociception was induced by intraplantar injection of 1.5% formalin (20 μL). Flinching behavior are showed in panel A in a temporal course and expresed as AUC in panel B. Licking behavior are showed in panel C in a temporal course and expresed as AUC in panel D. Vehicle, tramadol (100 mg/kg, *i.p.*, a positive control), **1** (100, 177, and 300 mg/kg, *i.p.*) and **12** (100, 177, and 300 mg/kg, *i.p.*) were administered 30 min before formalin. Data represent mean ± SEM (*n* = 6 per group). *p < 0.05 indicates significant differences vs vehicle (one-way ANOVA followed by Tukey’s test).

Hofmeisterin I (**1**) at 177 mg/kg reduced flinching behavior in a dose-dependent manner; however, these differences were not statistically significant compared with the vehicle (Figure 4A,B). In contrast, compound **12** (177 mg/kg) produced significant reductions, particularly in phase II (Figure 4A,B), demonstrating superior efficacy relative to the parent compound. To demonstrate a dose-dependent effect, licking behavior was evaluated (Figure 4C), and licking was observed in both phases I and II following vehicle administration. In this assay, the antinociceptive effect of tramadol was more pronounced, as formalin-induced licking behavior was almost completely abolished. Figure 4D shows that imide **12** has a clear dose-dependent antinociceptive effect on licking behavior.^25^ In this case, the zebrafish data, used as a preliminary screening tool, did not fully predict mammalian outcomes, likely reflecting species-specific differences in metabolism, absorption, and dose exposure.^8^

Based on these findings, imide **12** was selected for further studies to elucidate its possible mechanism of action (Figure 5). Selective antagonists were used to probe receptor involvement: naloxone (opioid), ketanserin (5-HT_2_), capsazepine (TRPV1), AM630 (CB_2_), and GW9662 (PPARγ).

**Figure 5:**
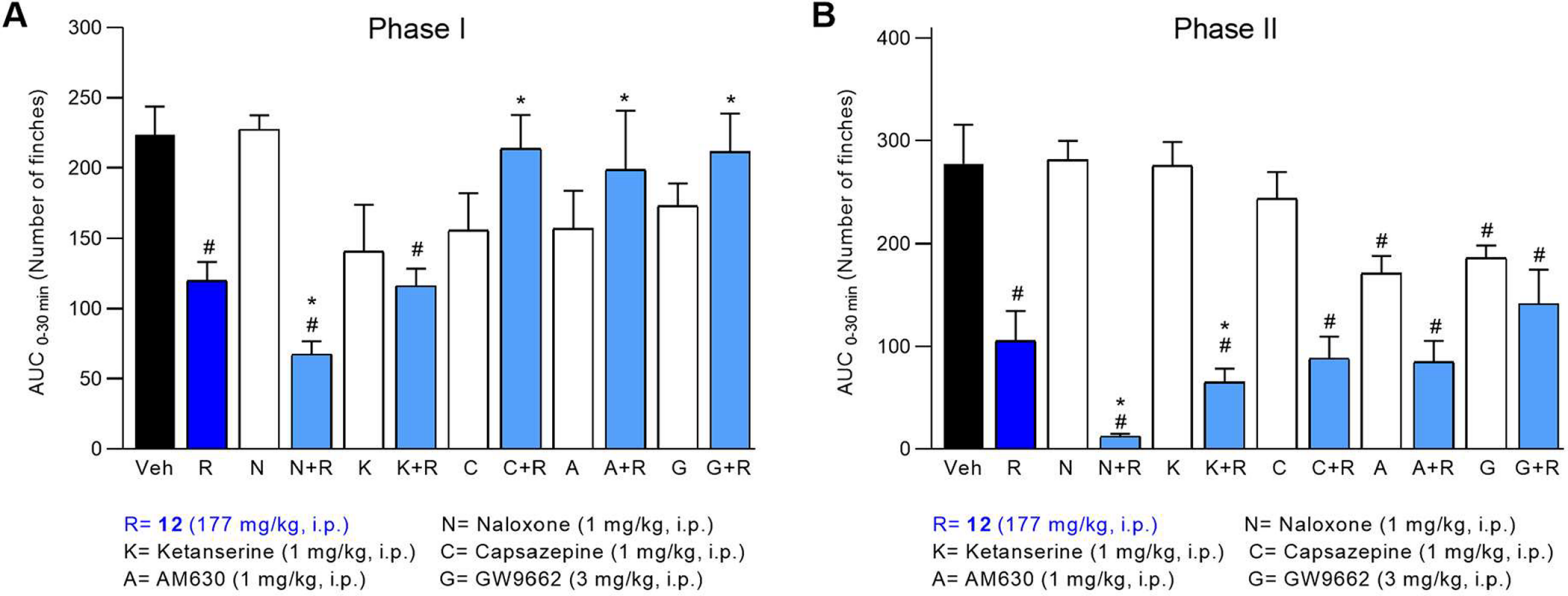
Antinociceptive mechanism of action of compound **12**. Nociception was induced by intraplantar injection of 1.5% formalin (20 μL). The flinching behavior is expressed as AUC, phase I on panel A and phase II on panel B. Vehicle, **12** (177 mg/kg, *i.p*.), naloxone (1 mg/kg, *i.p*), ketanserin (1 mg/kg, *i.p*.), capsazepine (1 mg/kg, *i.p.*), AM630 (1 mg/kg, *i.p.*), GW9662 (3 mg/kg, *i.p.*), were administered 15 min before **12** (177 mg/kg, *i.p*.). Data represent mean ± SEM (*n* = 6 per group). # *p* < 0.05 indicates significant differences vs vehicle and * indicates significant differences vs **12** (one-way ANOVA followed by Tukey’s test).

During Phase I (neurogenic pain), the antinociceptive effect of compound **12** was not blocked by naloxone, indicating that opioid receptors are not involved in its mechanism of action. On the contrary, a potentiation effect was observed, which is unexpected and requires further investigation.^26–28^ The lack of effect of ketanserin indicates that 5-HT_2_ receptors do not contribute to Phase I activity, while the partial reversal induced by capsazepine, AM630, and GW9662 in the early antinociceptive response. supports the involvement of TRPV1,^29^ CB_2_,^30^ and PPARγ signaling,^31^ respectively. TRPV1 and CB2 receptors exert a tonic inhibitory effect on formalin-induced inflammatory pain.^20,21^ PPARγ receptors are ligand-activated transcription factors that regulate the expression of many genes, and PPARγ can influence pain. Agonists of these receptors are also involved in anti-inflammatory activity.^22^

In Phase II (inflammatory/central sensitization), naloxone again increased the antinociceptive effect of compound **12**. This unexpected effect warrants further investigation, supporting the conclusion that opioid pathways do not mediate its analgesic action in either phase. The additional potentiation observed with ketanserin during Phase II suggests a context-dependent serotonergic modulation, in which blockade of 5-HT_2_ receptors may eliminate facilitatory serotonergic tone, thereby enhancing alternative analgesic pathways. This interpretation aligns with previous findings showing that ketanserin potentiates antinociception induced by nonopioid compounds in inflammatory pain models.^32,33^

Unlike in Phase I, TRPV1’s role was minimal during Phase II, as neither capsazepine alone nor its combination with compound **12** significantly affected nociceptive behavior. CB_2_ and PPARγ receptors displayed different patterns of modulation in this phase: although both antagonists reduced flinching when used alone, their combination with compound **12** did not consistently diminish its antinociceptive effect, suggesting that these pathways might influence baseline inflammatory nociception rather than directly mediate the compound’s activity during Phase II.

Overall, these findings suggest that compound **12** produces antinociception through multiple nonopioid receptor systems, with the relative contribution of TRPV1, CB_2_, PPARγ, and serotonergic signaling varying across nociceptive phases. Although these receptors are linked to nociceptive pathways, their specific role in mediating the effects of compound **12** remains preliminary and requires further investigation to clarify their individual mechanisms of action and potential pharmacological interactions that contribute to the antinociceptive effect. Future studies involving the central nervous system, including locomotor activity and motor coordination tests (such as open-field or rotarod tests), will be crucial to completely rule out sedation or motor-related influences on the observed antinociceptive responses.

### 3.5 Docking Analysis

Compound **12** demonstrated significant antinociceptive activity in both zebrafish and murine models, with a more pronounced effect during the inflammatory phase of the formalin test. This phase-dependent activity suggests involvement of mechanisms associated with central sensitization and inflammatory pain processing rather than acute neurogenic nociception. The enhanced efficacy observed in the presence of ketanserin, together with the absence of naloxone antagonism, indicates that serotonergic and opioid pathways may contribute to the effects in a modulatory rather than a primary manner.

To explore this possibility at the molecular level, docking studies were conducted using serotonergic 5-HT_2__A_, 5-HT_2__B_, and 5-HT_2__C_ receptors, as well as the μ-opioid receptor (Figure 6; Table 1; Figures S27 and S28). Docking to the μ-opioid receptor revealed a predicted binding energy of −7.6 kcal/mol for compound **12**, which was notably weaker than that observed for naloxone (−9.0 kcal/mol).^32^ This finding is consistent with the pharmacological data, which show that naloxone did not abolish the antinociceptive effect, suggesting that μ-opioid receptor engagement is unlikely to be the dominant mechanism of action.

**Figure 6:**
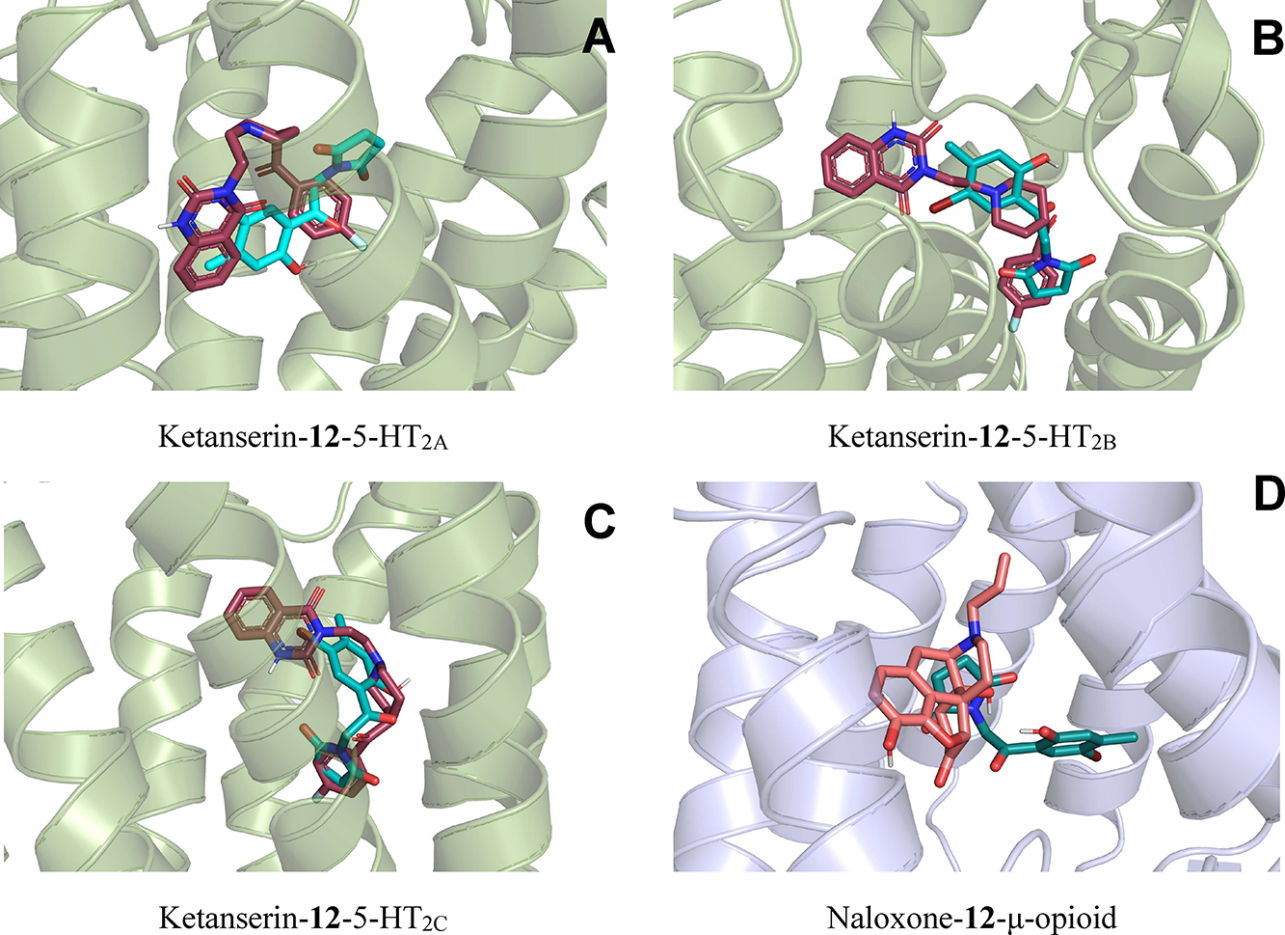
3D schemes for compound **12** (cyan sticks) and ketanserin (red sticks) with the 5-HT_2A_ (panel A), 5-HT_2B_ (panel B), 5-HT_2C_ (panel C); naloxone (orange sticks) with μ-opioid (panel D) receptors. Created with PyMol software.

**Table 1:** Binding Energies of the Docked Compound **12** in the Different Receptors

	binding energy (kcal/mol)			
compound	5-HT_2A_	5-HT_2B_	5-HT_2C_	μ-opioid
compound **12**	–7.4	–7.9	–8.2	–7.6
ketanserin	–9.6	–11.3	–10.3	
naloxone				–9.0

Because ketanserin is a preferential 5-HT_2A_ antagonist but can also interact with other members of the 5-HT_2_ receptor family, in silico screening against all three subtypes was conducted to offer a more comprehensive understanding of potential serotonergic interactions. Compound **12** exhibited moderate predicted binding affinities across the three 5-HT_2_ receptor subtypes, ranging from −7.4 to −8.2 kcal/mol (Table 1), with the most favorable interaction observed for the 5-HT_2C_ receptor. These values were consistently weaker than those obtained for ketanserin (−9.6 to −11.3 kcal/mol, Table 1).^33^

Taken together, the docking results predicted a moderate interaction between compound **12** and the 5HT_2C_ receptor, which partially inhibits nociception in phase 2 of the formalin test. These interactions are best interpreted as complementary to other mechanisms identified through pharmacological assays, including those involving the TRPV1, CB_2_, and PPARγ pathways. As such, the antinociceptive profile of compound **12** likely arises from a multifactorial mechanism that integrates peripheral and central modulation, rather than from engagement of a single-target receptor.

## 4 Conclusions

In this study, an optimized synthetic route for hofmeisterin I (**1**) was successfully developed, resulting in significantly higher yields and shorter reaction times, allowing for its biological evaluation. Although two halogenated derivatives were synthesized efficiently, they did not exhibit improved antinociceptive activity and showed increased toxicity, suggesting that halogen substitution on the aromatic ring of hofmeisterin negatively impacts both efficacy and toxicity. The acetyl derivative of compound **1** demonstrated enhanced antinociceptive potency compared to hofmeisterin I; however, its toxicity at higher doses limits its therapeutic potential. *In vivo* evaluation showed that **1** had moderate antinociceptive activity, lower than that of the reference drug, tramadol. Notably, the nitrogen-containing imide analogue **12**, a new chemical entity, despite containing bromine, displayed superior antinociceptive efficacy and a favorable safety profile in both zebrafish and murine models. Mechanistic studies revealed that the antinociceptive action of compound **12** is mediated by multiple nonopioid pathways, including TRPV1, CB_2_, and PPARγ receptors, as well as the serotonergic pathway. The docking results align with behavioral findings showing that ketanserin and naloxone enhanced the antinociceptive effect of compound **12** in both phases of the formalin test in mice. Overall, these findings identify compound **12** as a promising candidate for further development as an antinociceptive agent.

## Supplementary Material









## Notes

^1^H and ^13^C NMR of all compounds synthesized in this work (PDF)
X-ray structures of compounds **1** (CIF)
X-ray structures of compounds **9b** (CIF)
X-ray structures of compounds **12** (CIF)
