# Supplementary material for: Comparative Antinociceptive Evaluation of Hofmeisterin I and Analogues from Hofmeisteria schaffneri in Zebrafish and Mice
Source: ACS Omega. 2026 Apr 11;11(15):23469–79. doi: 10.1021/acsomega.6c01037 (PMC13103808; doi:10.1021/acsomega.6c01037)
Supplement: Supplementary file 1 [file ao6c01037_si_001.pdf]

Comparative Antinociceptive Evaluation of Hofmeisterin I and Analogues from *Hofmeisteria schaffneri* in Zebrafish and Mice

Sebastián Martínez Flores,[a,#] Gabriela García-Marín,[a,#] María Guadalupe Martínez-Villarreal,[a] Manuel López-Ortiz,[b] Manuel Eduardo Rangel-Grimaldo,[c] Simón Hernández Ortega,[c] Myrna Déciga-Campos,[d*] and Rachel Mata[a**]

[a] Departamento de Farmacia, Facultad de Química, Universidad Nacional Autónoma de México, Ciudad de México 04510, México

[b] Laboratorio de Síntesis Orgánica, UMIEZ Facultad de Estudios Superiores Zaragoza, Universidad Nacional Autónoma de México, Campus II, Ciudad de México 09230, México

[c] Laboratorio de Difracción de Rayos X and Departamento de Productos Naturales, Instituto de Química, Universidad Nacional Autónoma de México, Ciudad de México 04510, México

[d] Sección de Estudios de Posgrado e Investigación, Escuela Superior de Medicina, Instituto Politécnico Nacional, Ciudad de México, 11340, México

* Corresponding author.

** Corresponding author.

E-mail addresses: rachel@unam.mx, mdeciga@ipn.com

Table of contents:

1. Table 1. Optimization of hydrogenolysis S3
2. NMR spectra S4-S16
3. Figure S27 S17
4. Figure S28 S18

Table S1. Optimization of hydrogenolysis.

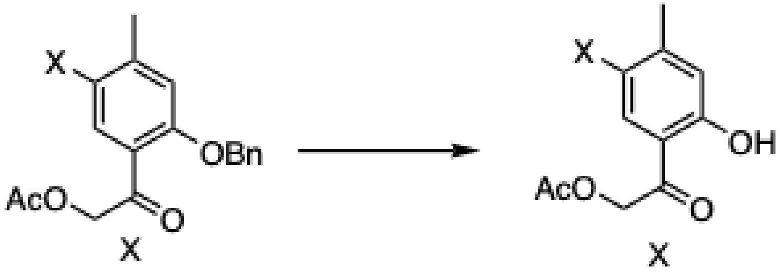

8a, X=Br
8b, X=Cl

8a, X=Br
8b, X=Cl

Compound	Catalyst	Temperature (°C)	Time (h)	Solvent	Yield (%)
**8a**	Pd/C (10 %)	r.t	2	AcOEt	Trace
**8a**	Pd/C (10 %)	60	18	AcOEt	Trace
**8a**	Pd/C (10 %)	60	2	AcOH	Trace
**8a**	Pd/C (10 %)	60	18	AcOH	Trace
**8a**	$Pd(OH)_2/C$ (20 %)	r.t	2	AcOEt	Trace
**8a**	$Pd(OH)_2/C$ (20 %)	60	6	AcOEt	Trace
**8a**	PtO_2	r.t	2	AcOH	0
**8a**	PtO_2	r.t	2	AcOEt	98
**8a**	PtO_2	60	2	AcOEt	98
**8b**	PtO_2	r.t	2	AcOEt	86

Al reactions were carried out with 10 % (w/w) of catalyst, 100 mg of compound and H_2 (60 lb/inch^2).

2. NMR spectra

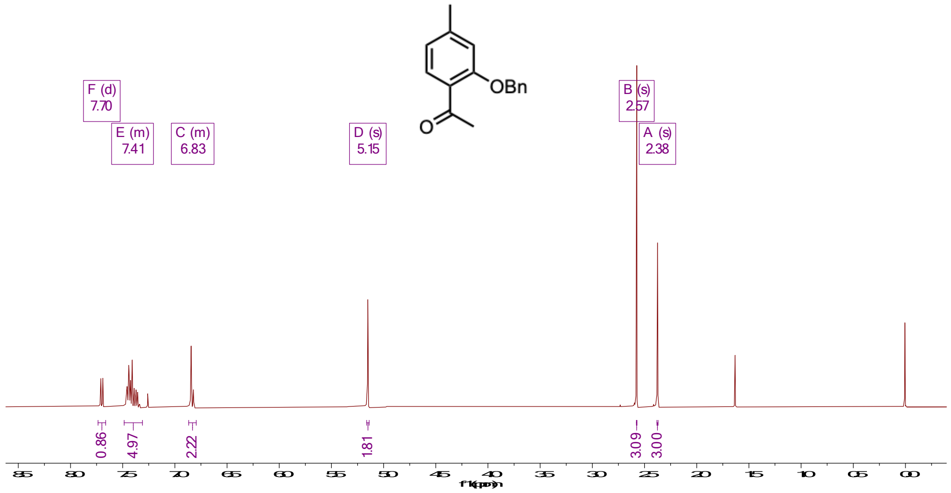

Figure S1. ^{1}H NMR (400 MHz, $CDCl_3$) of **5**.

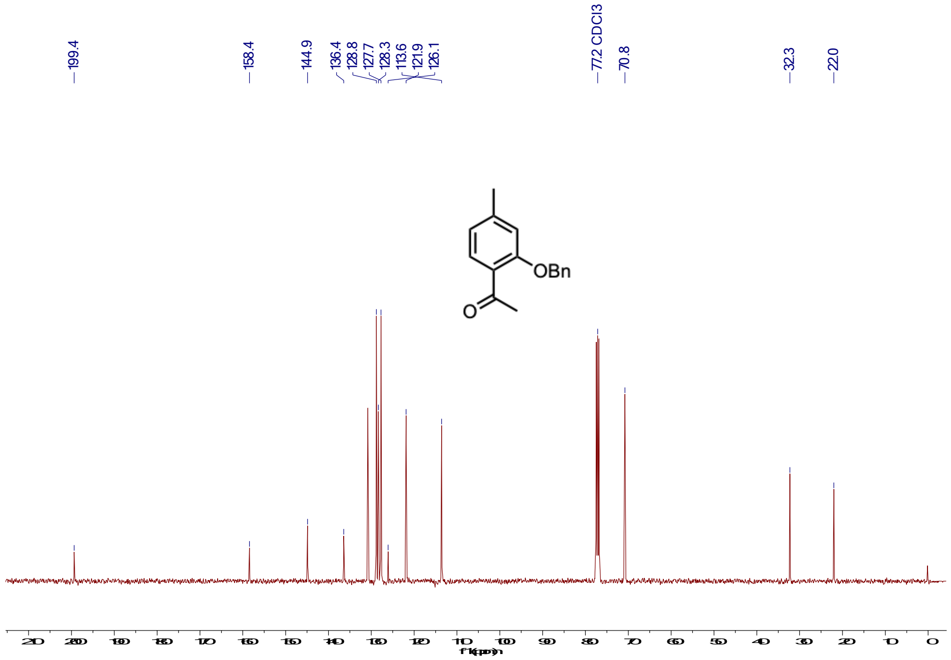

Figure S2. ^{13}C NMR (100 MHz, $CDCl_3$) of **5**.

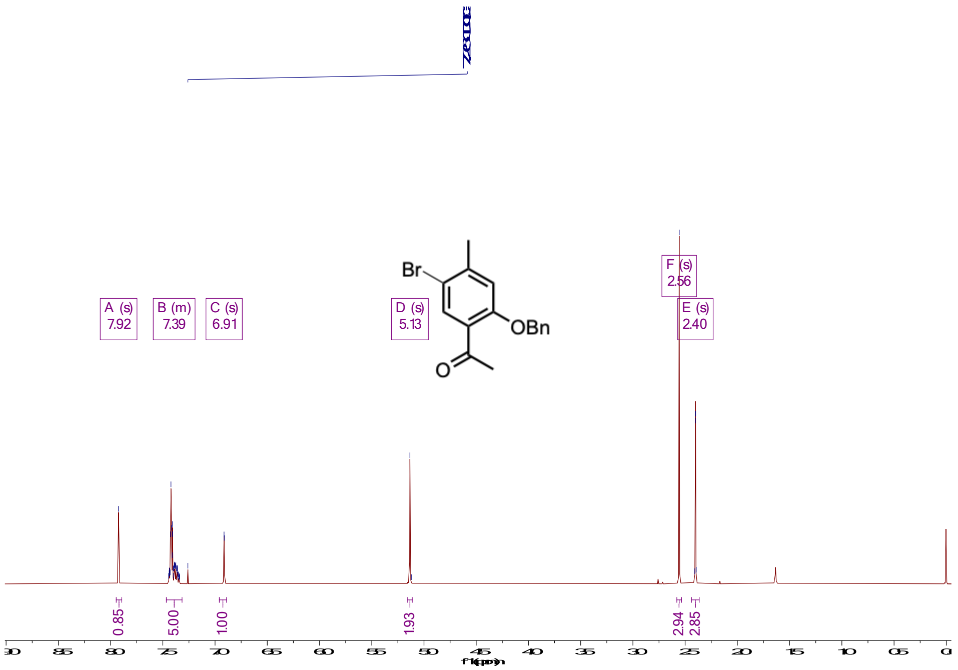

Figure S3. ^{1}H NMR (400 MHz, $CDCl_3$) of **6a**.

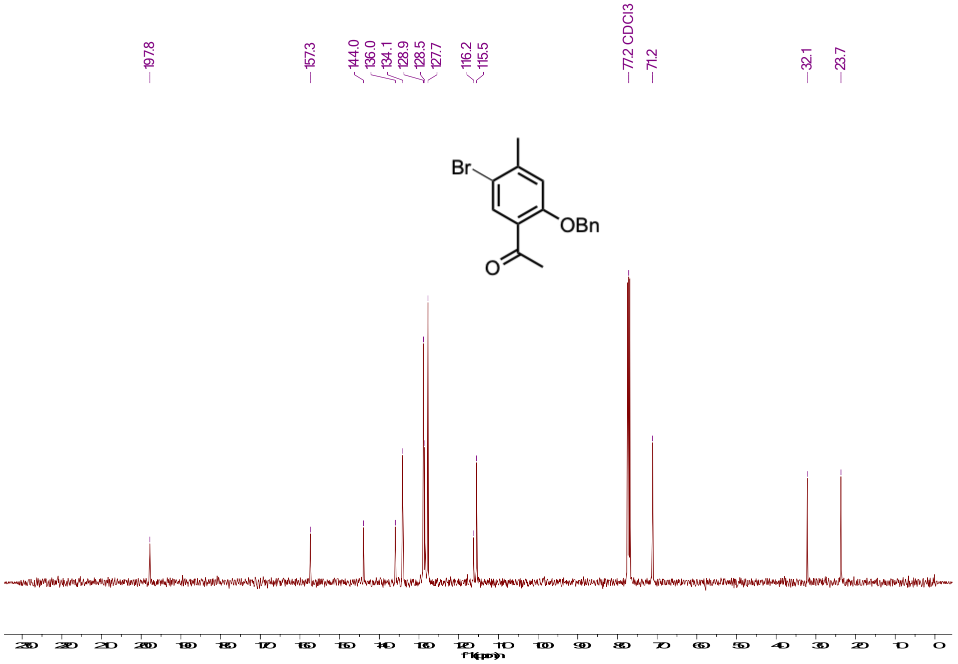

Figure S4. ^{13}C NMR (100 MHz, $CDCl_3$) of **6a**.

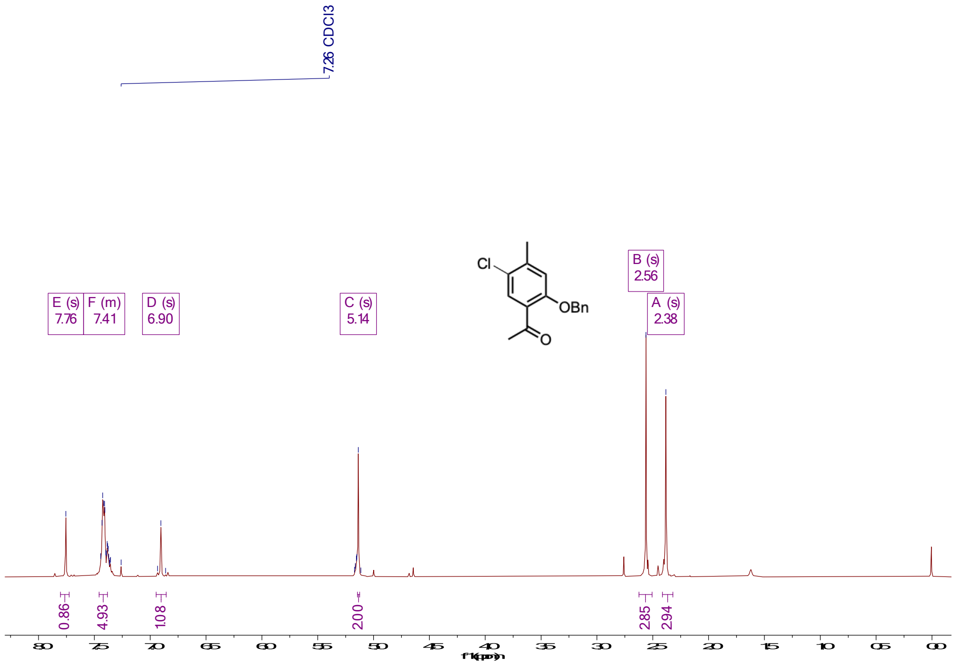

Figure S5. ^{1}H NMR (300 MHz, $CDCl_3$) of **6b**.

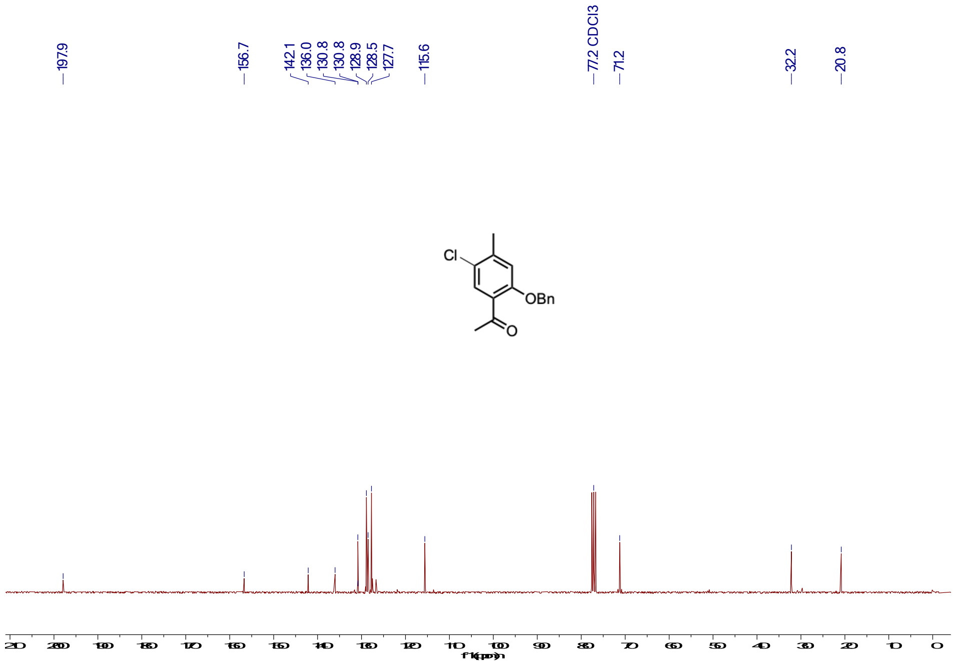

Figure S6. ^{13}C NMR (75 MHz, $CDCl_3$) of **6b**.

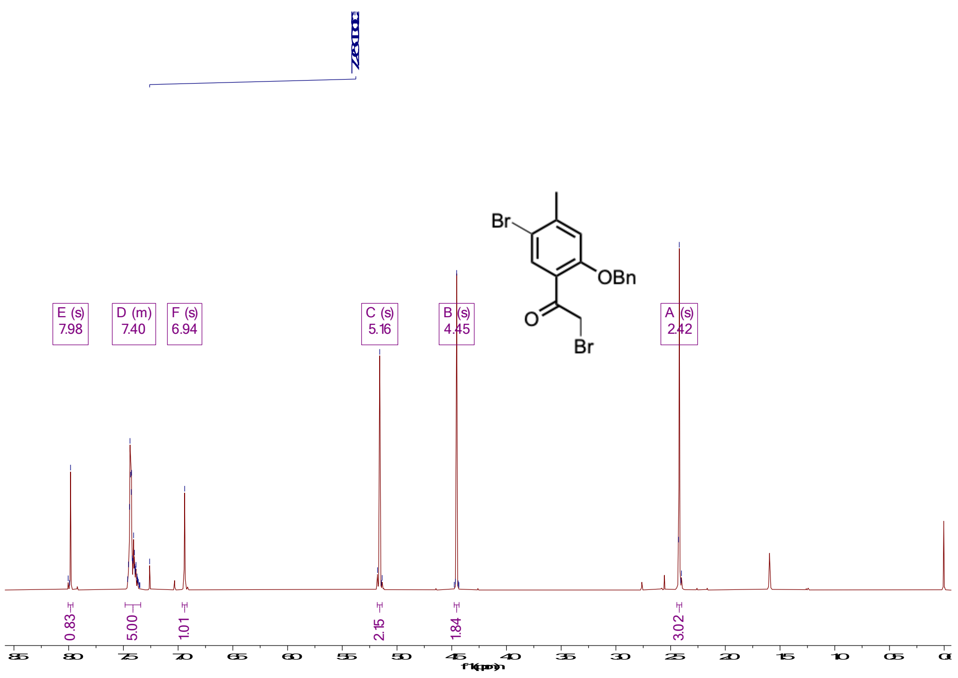

Figure S7. ^{1}H NMR (400 MHz, $CDCl_3$) of **7a**.

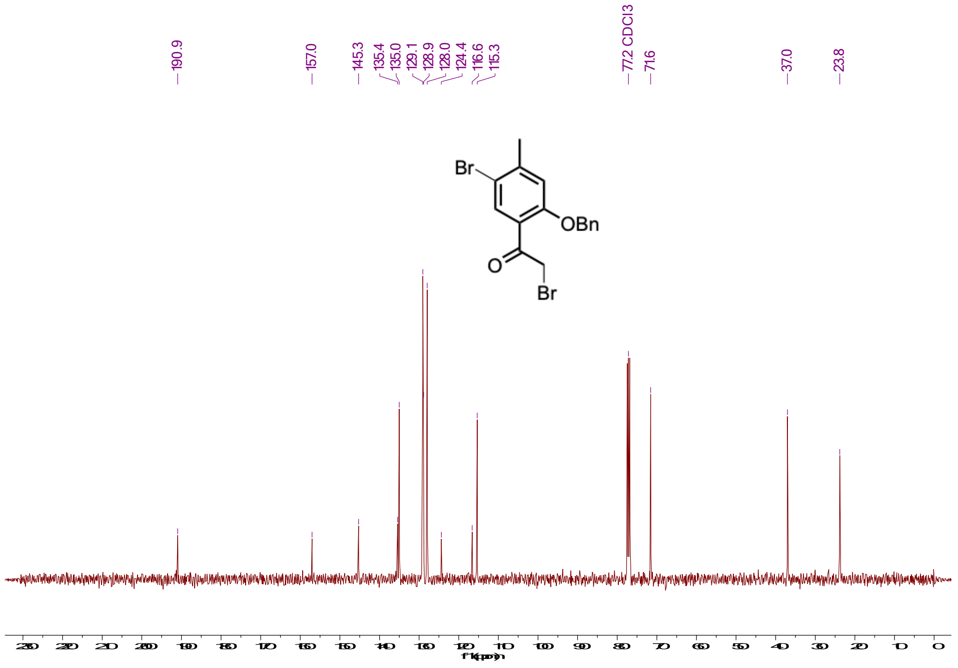

Figure S8. ^{13}C NMR (100 MHz, $CDCl_3$) of **7a**.

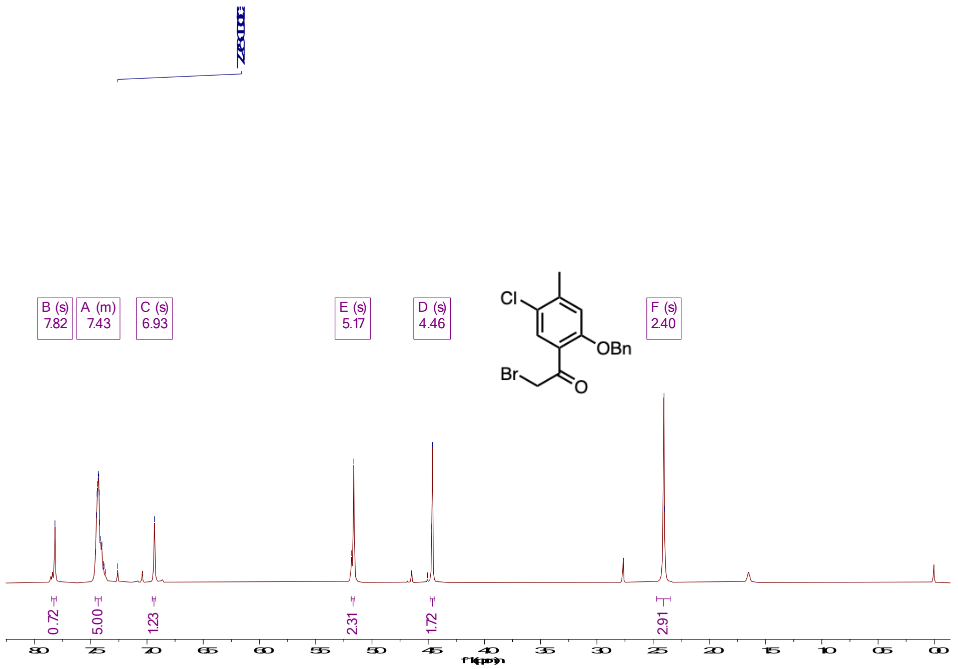

Figure S9. ^{1}H NMR (300 MHz, $CDCl_3$) of **7b**.

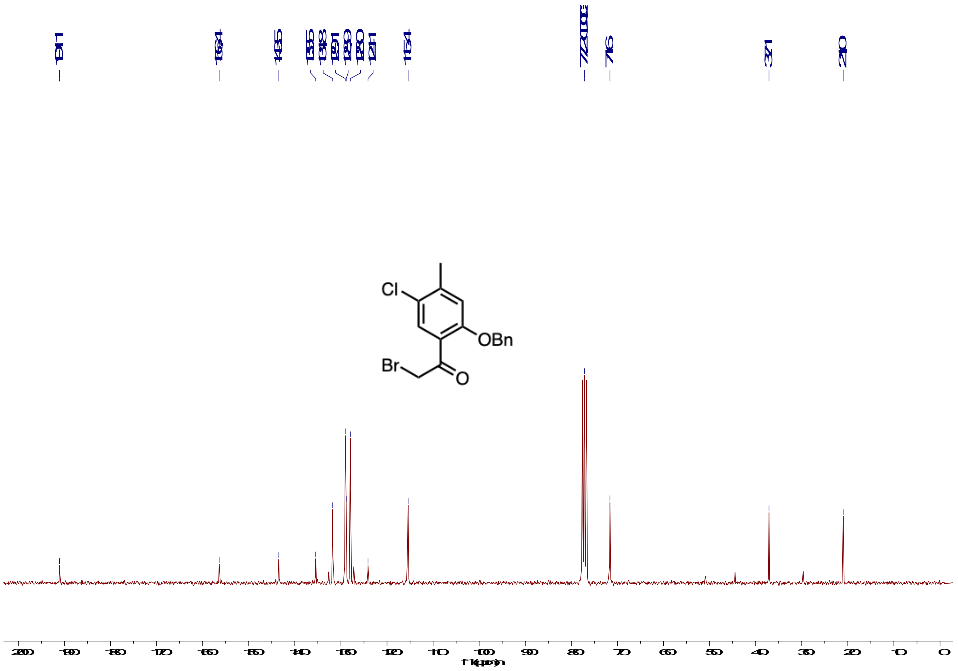

Figure S10. ^{13}C NMR (75 MHz, $CDCl_3$) of **7b**.

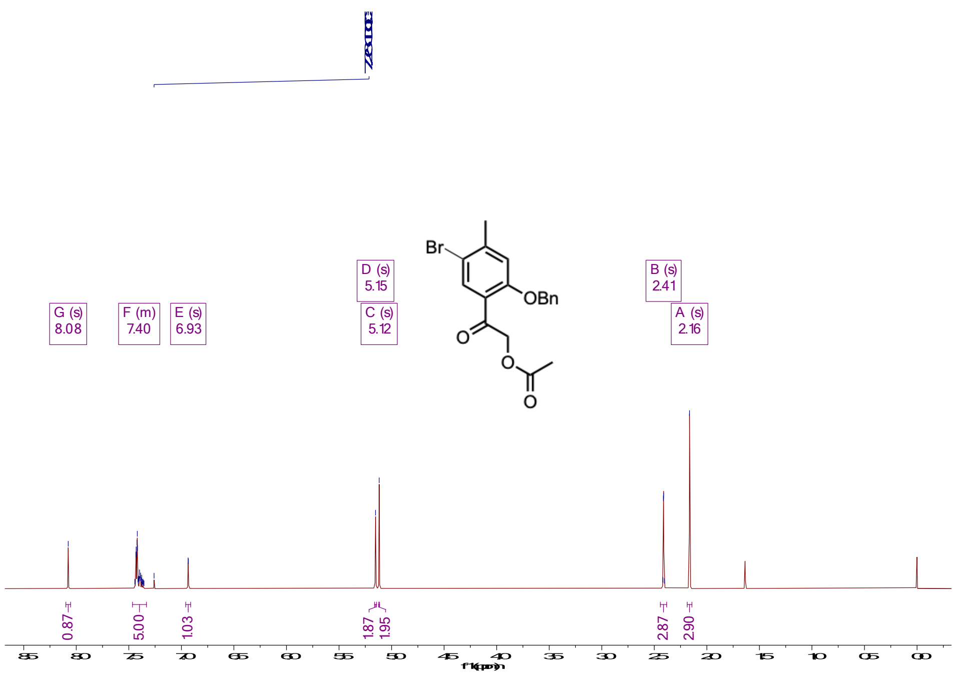

Figure S11. ^{1}H NMR (400 MHz, $CDCl_3$) of **8a**.

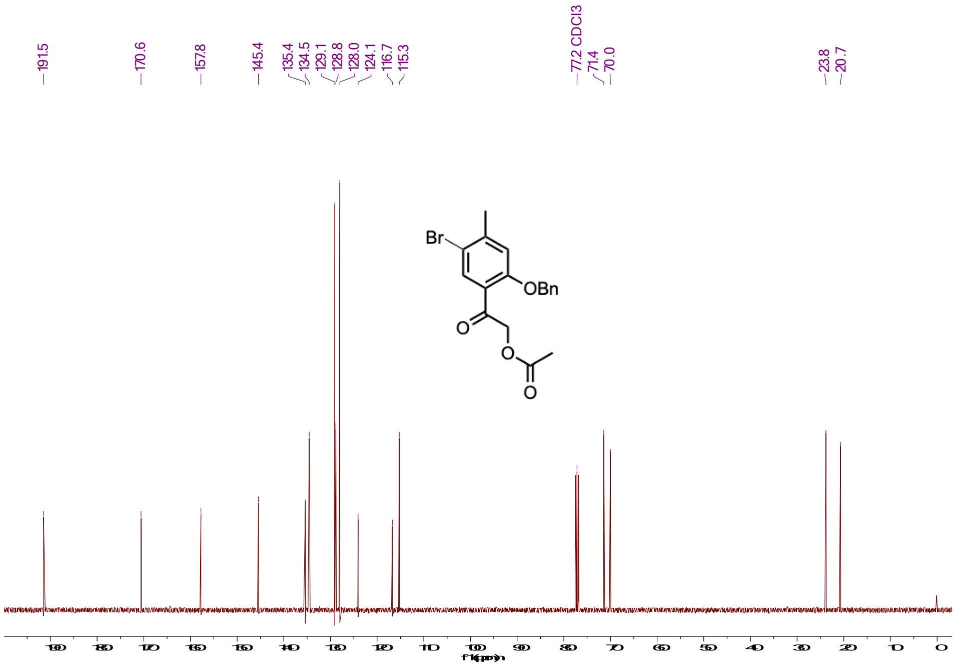

Figure S12. ^{13}C NMR (100 MHz, $CDCl_3$) of **8a**.

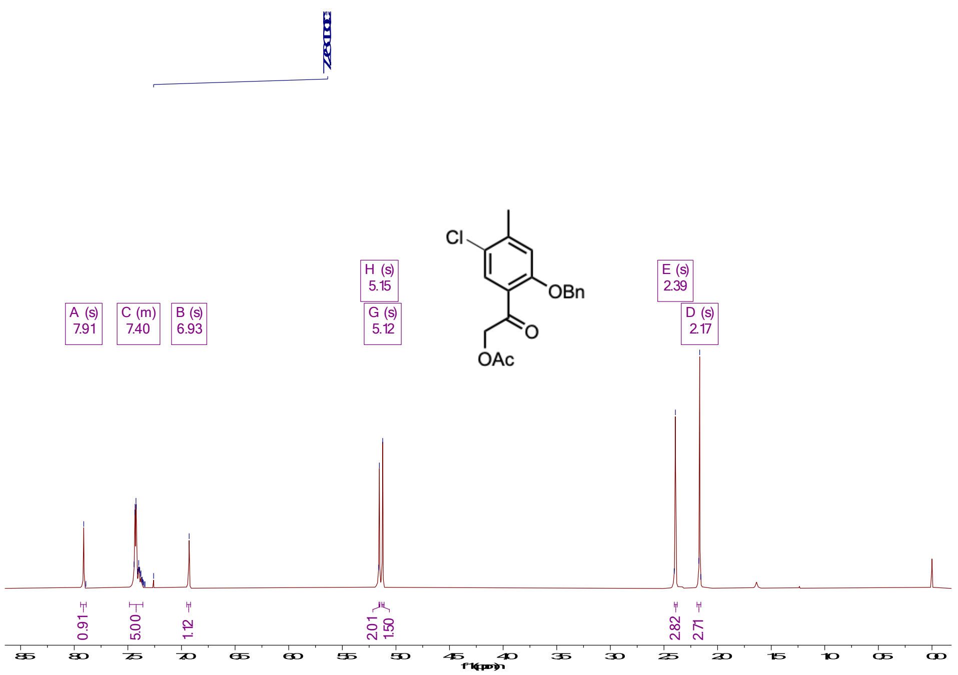

Figure S13. ^{1}H NMR (400 MHz, $CDCl_3$) of **8b**.

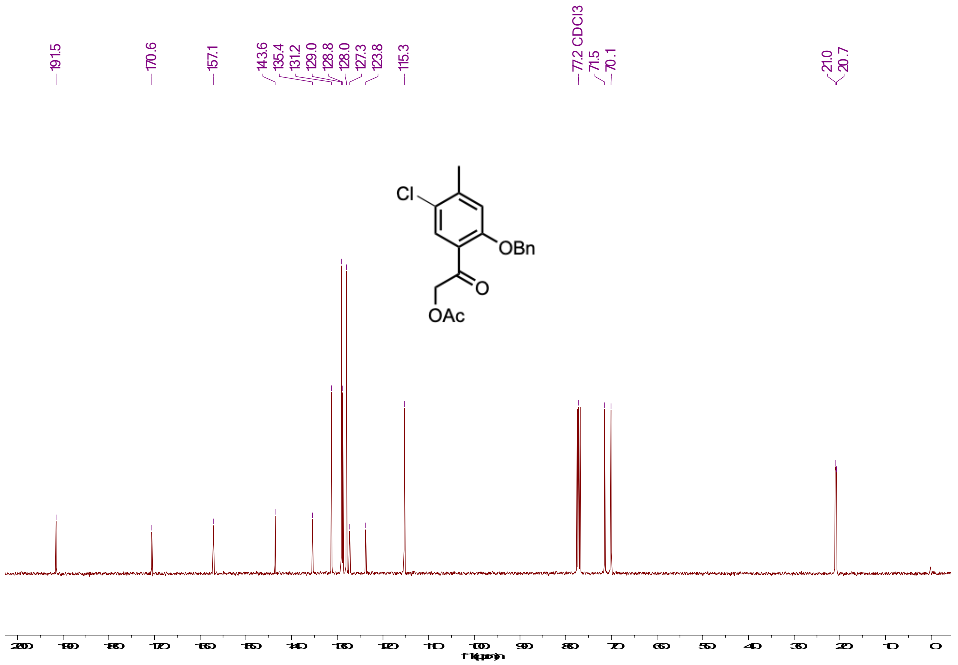

Figure S14. ^{13}C NMR (100 MHz, $CDCl_3$) of **8b**.

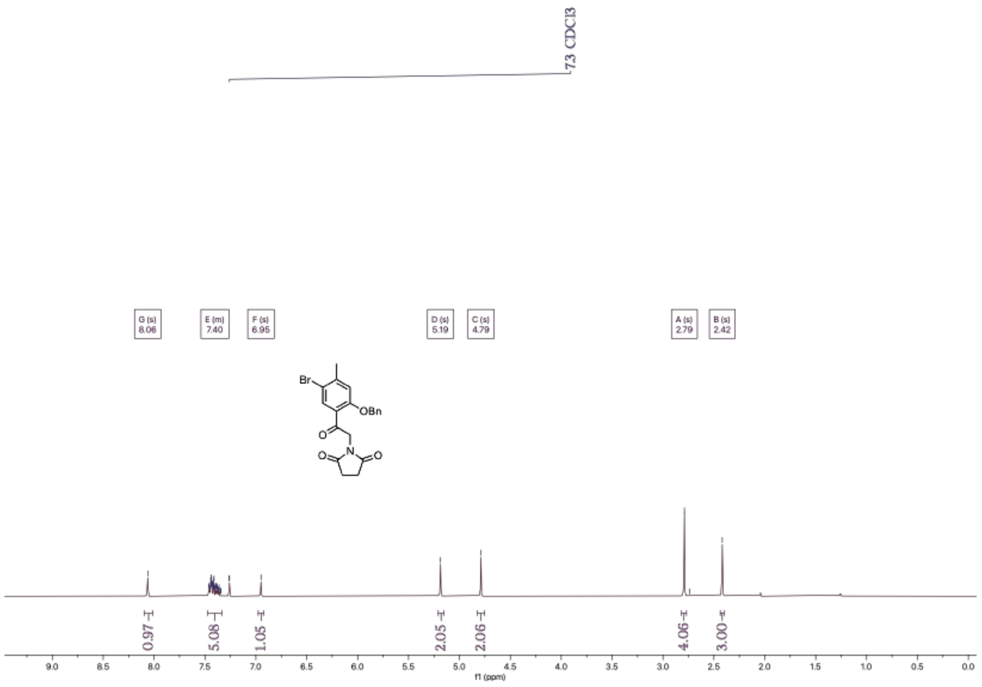

Figure S15. ^{1}H NMR (400 MHz, $CDCl_3$) of **11**.

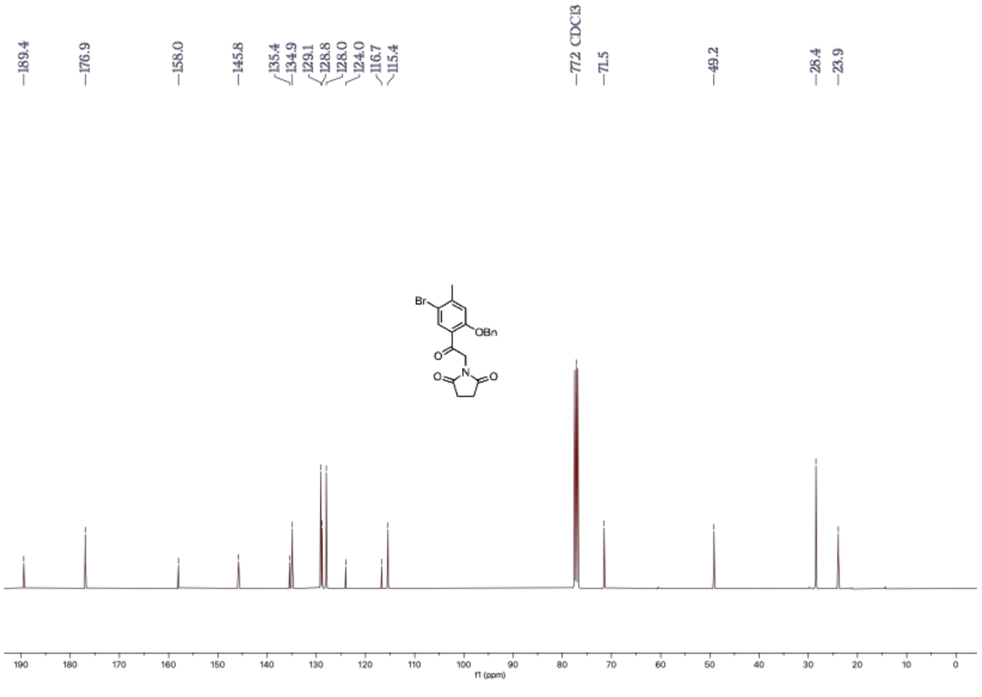

Figure S16. ^{13}C NMR (100 MHz, $CDCl_3$) of **11**.

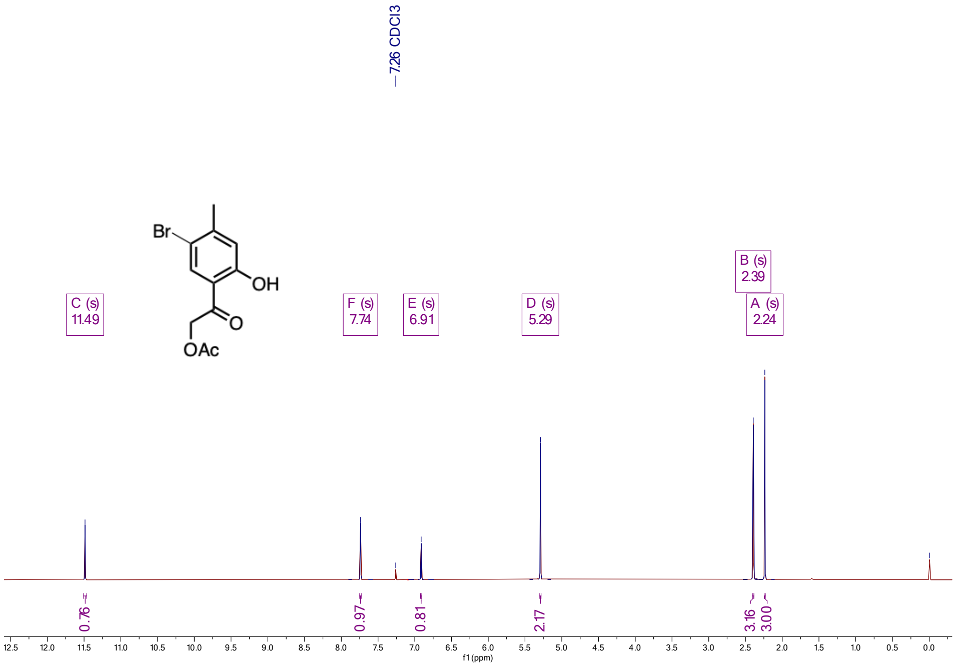

Figure S17. ¹H NMR (600 MHz, $CDCl_3$) of **9a**.

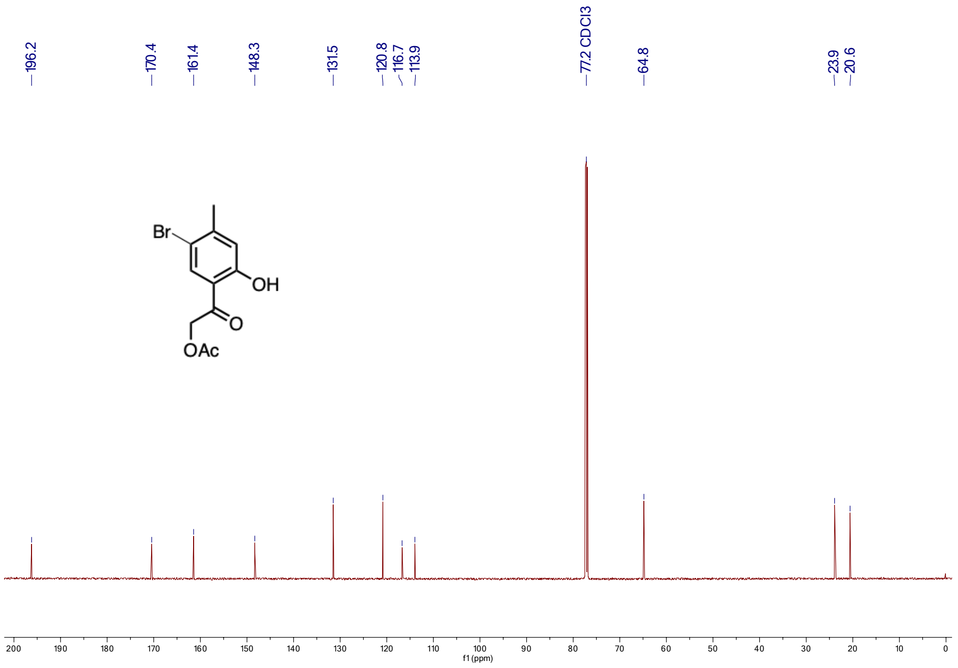

Figure S18. ¹³C NMR (150 MHz, $CDCl_3$) of **9a**.

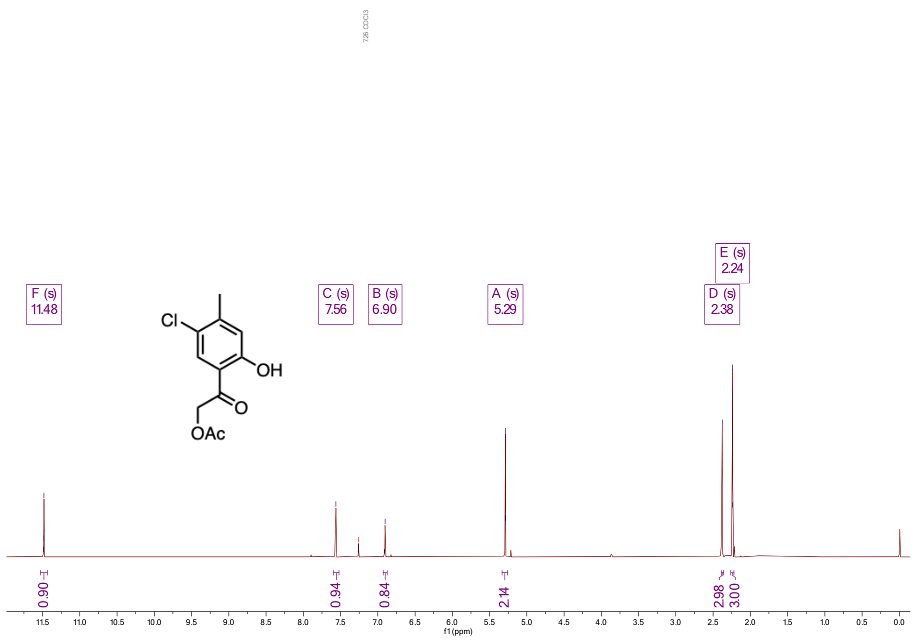

Figure S19. ^{1}H NMR (600 MHz, $CDCl_3$) of **9b**.

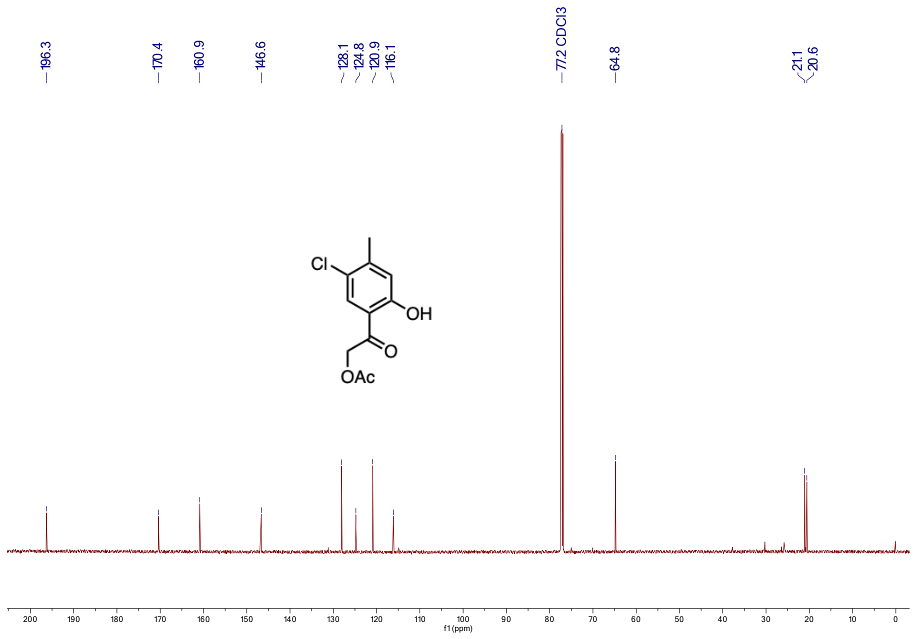

Figure S20. ^{13}C NMR (150 MHz, $CDCl_3$) of **9b**.

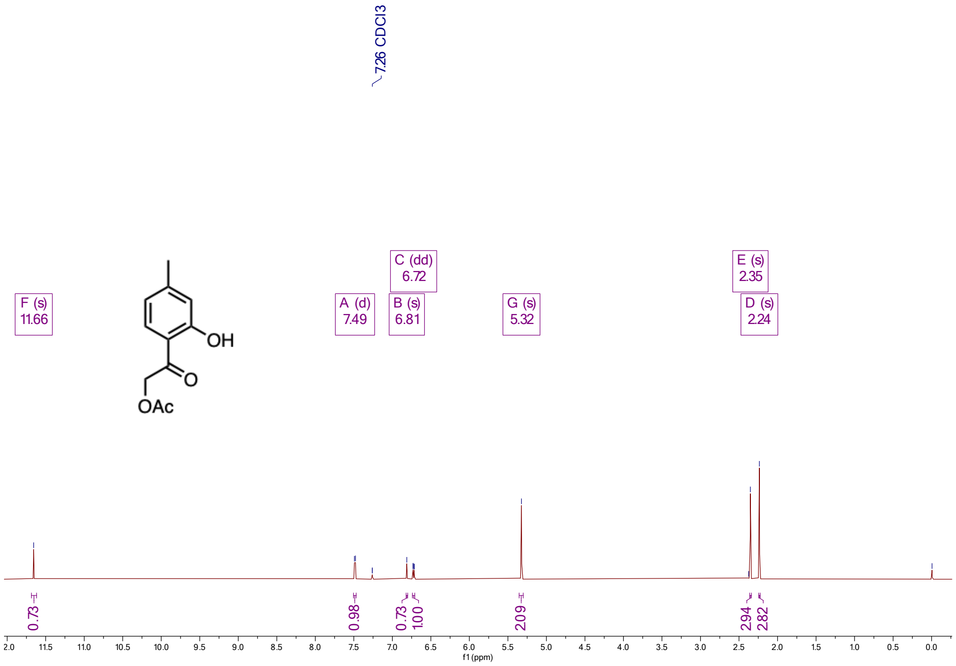

Figure S21. ^{1}H NMR (600 MHz, $CDCl_3$) of **1**.

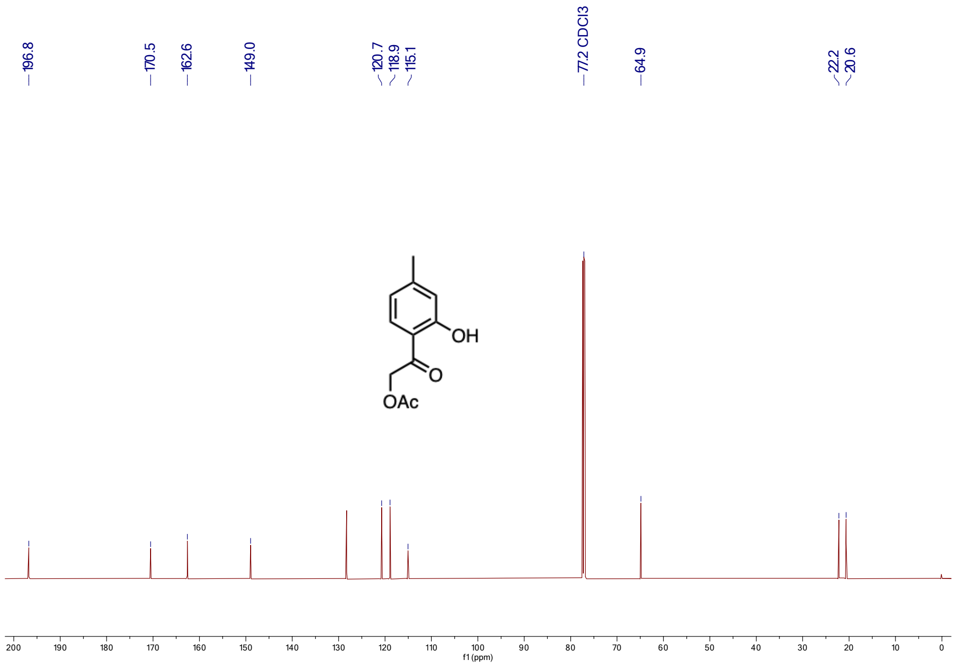

Figure S22. ^{13}C NMR (150 MHz, $CDCl_3$) of **1**.

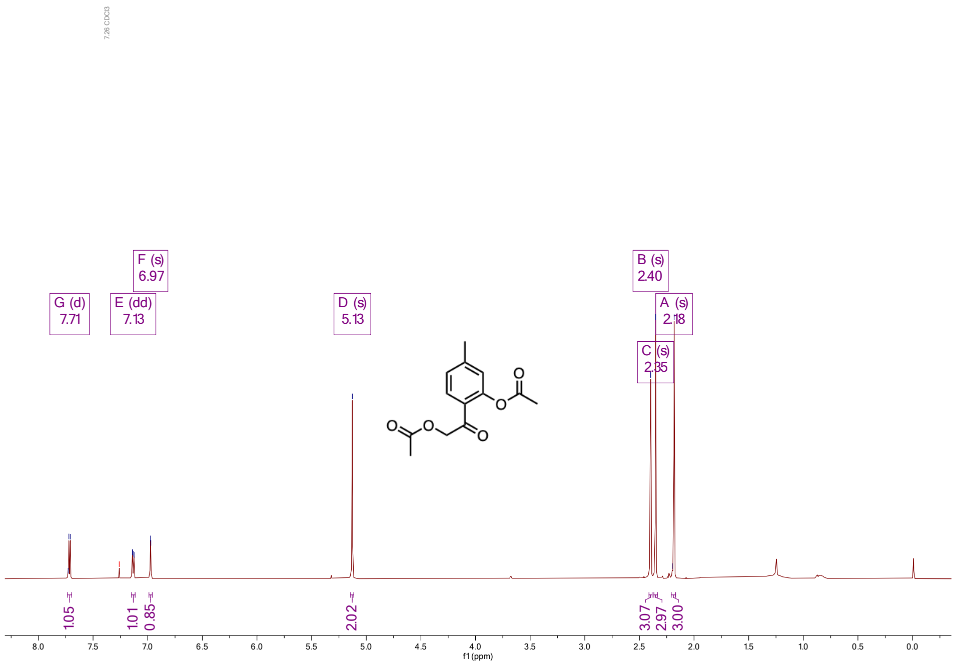

Figure S23. ^{1}H NMR (600 MHz, $CDCl_3$) of **10**.

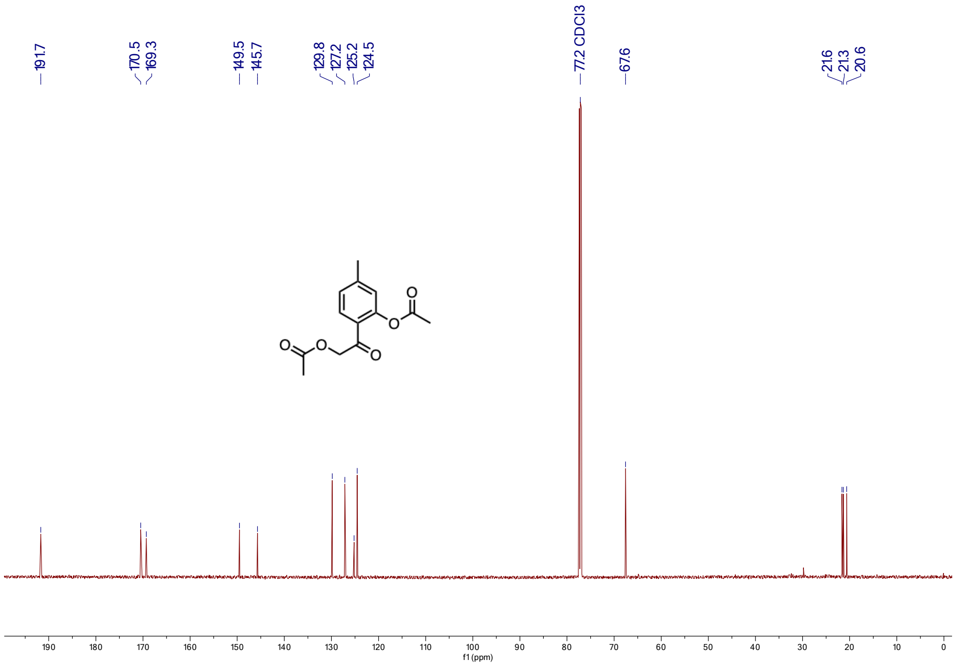

Figure S24. ^{13}C NMR (150 MHz, $CDCl_3$) of **10**.

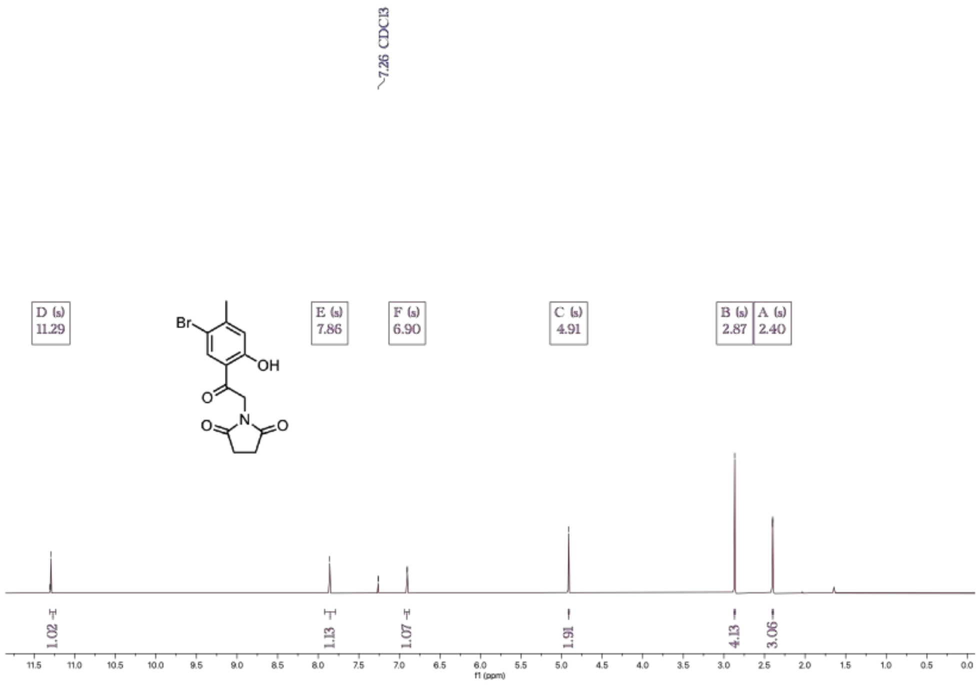

Figure S25. ^{1}H NMR (400 MHz, $CDCl_3$) of **12**.

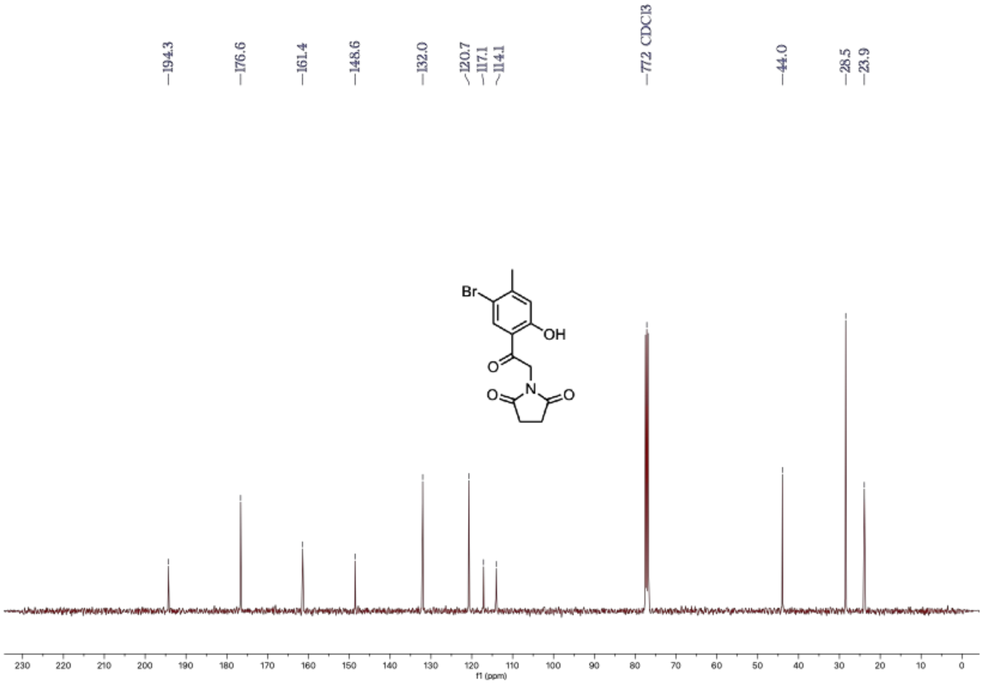

Figure S26. ^{13}C NMR (100 MHz, $CDCl_3$) of **12**.

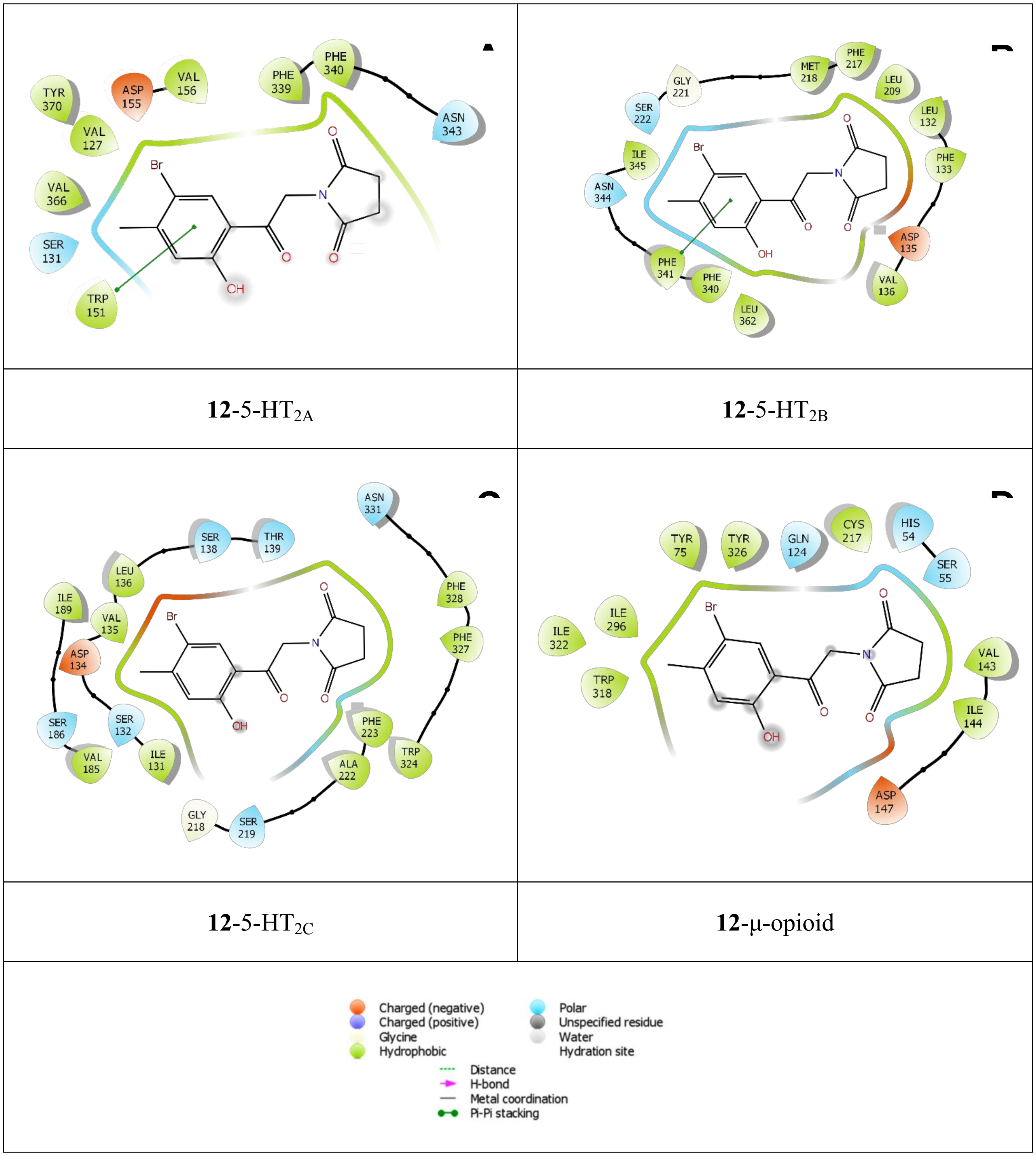

Figure S27. Schematic 2D plots of intermolecular interactions observed for compound **12** with the 5-HT$_{2A}$ (panel A), 5-HT$_{2B}$ (5TVN; panel B), 5-HT$_{2C}$ (6BQH; panel C) and μ-opioid (5C1M; panel D) receptors. Created with Maestro software (2018-4).

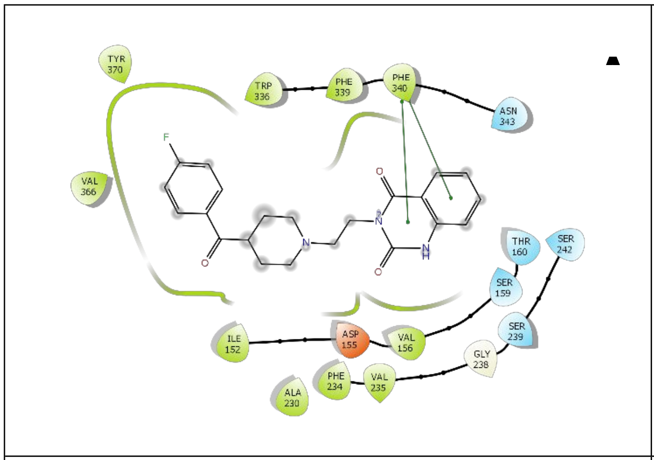

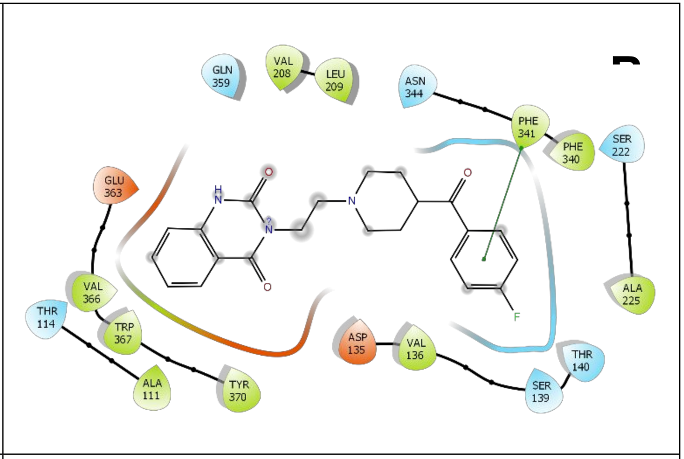

ketanserin -5-HT_{2A}

ketanserin -5-HT_{2B}

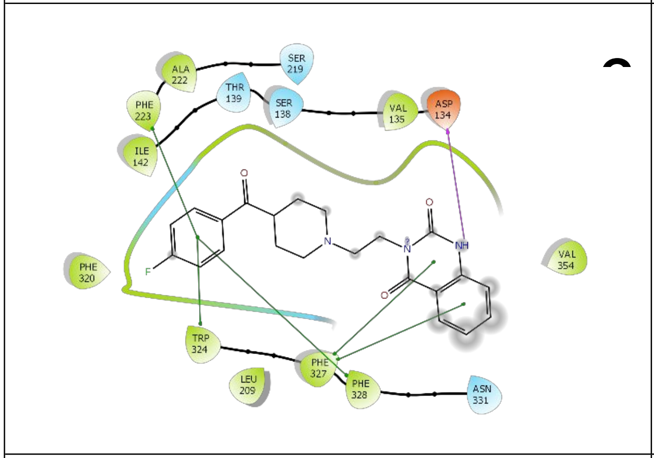

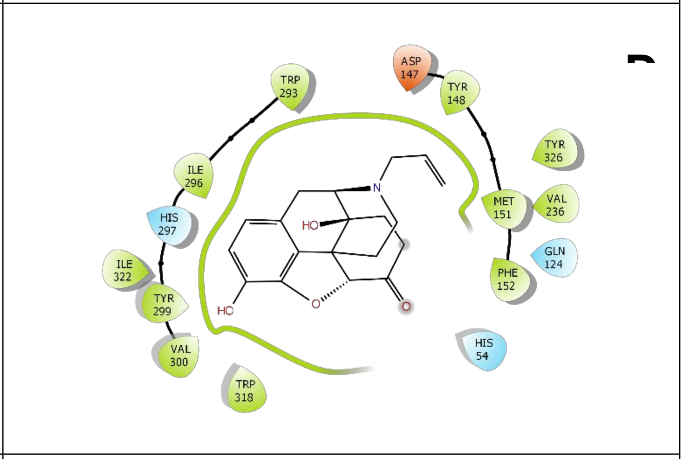

ketanserin -5-HT_{2C}

naloxone -μ-opioid

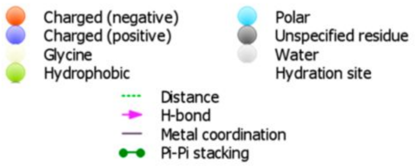

Figure S28. Schematic 2D plots of intermolecular interactions observed for ketanserin with the 5-HT_{2A} (panel A), 5-HT_{2B} (5TVN; panel B), 5-HT_{2C} (6BQH; panel C), and naloxone with the μ-opioid (5C1M; panel D) receptors. Created with Maestro software (2018-4).
